# Lineage-restricted genomic islands drive rhizosphere niche specialization in *Pseudomonas capeferrum*

**DOI:** 10.1093/ismeco/ycag231

**Published:** 2026-08-23

**Authors:** Miguel A López-Carrasco, Gabriela Purtschert-Montenegro, Marta Pinto-Carbo, Gerardo Cárcamo-Oyarce, Ratchara Kalawong, Leo Eberl

**Affiliations:** Department of Plant and Microbial Biology, University of Zurich, 8008 Zurich, Switzerland; Department of Plant and Microbial Biology, University of Zurich, 8008 Zurich, Switzerland; Department of Plant and Microbial Biology, University of Zurich, 8008 Zurich, Switzerland; Department of Plant and Microbial Biology, University of Zurich, 8008 Zurich, Switzerland; Department of Plant and Microbial Biology, University of Zurich, 8008 Zurich, Switzerland; Department of Plant and Microbial Biology, University of Zurich, 8008 Zurich, Switzerland

**Keywords:** *Pseudomonas* sp, genomic islands, plant growth–promoting rhizobacteria, horizontal gene transfer, biocontrol activity

## Abstract

In the rhizosphere, plant growth–promoting rhizobacteria play a pivotal role in plant health by efficiently colonizing roots and enhancing growth through diverse direct and indirect mechanisms. Many of these beneficial traits are associated with genomic islands (GEIs) acquired via horizontal gene transfer, yet their contribution to rhizosphere fitness remains poorly understood. Here, we report the whole-genome sequencing and comprehensive analysis of *Pseudomonas putida* strain IsoF, a highly efficient root colonizer originally isolated from the tomato rhizosphere that induces systemic resistance in plants. Phylogenomic analysis reclassified IsoF within the *P. capeferrum* group and revealed an extensive repertoire of GEIs encoding candidate functions to rhizosphere adaptation and plant growth promotion. Notably, IsoF harbours a unique combination of three GEIs absent in closely related strains. Phenotypic characterization and rhizosphere persistence assays using defined deletion mutants demonstrated that these islands significantly contribute to IsoF fitness and competitiveness in the rhizosphere. Together, our findings establish GEIs as key determinants of ecological success in beneficial rhizobacteria and position IsoF as a promising candidate for sustainable biocontrol applications.

## Introduction

Plant growth–promoting rhizobacteria (PGPR) are beneficial microbes inhabiting the rhizosphere, the soil fraction in contact with plant roots and influenced by their exudates. PGPR can enhance plant growth via a range of direct and indirect mechanisms, including bioremediation by degrading harmful pollutants or increasing nutrient bioavailability in the soil [1, 2]. They also suppress plant pathogens through competitive mechanisms, including the production of antibiotics, bacteriocins, or contact-dependent antagonism mediated by secretion systems [3–5]. In addition, various bacterial components such as flagella, lipopolysaccharides (LPS), and siderophores can trigger induced systemic resistance (ISR), priming the immune response of plants against phytopathogens, including bacteria, fungi, and viruses [2, 4].

The *Pseudomonas* group stand out as dominant PGPRs in the rhizosphere, displaying great metabolic diversity and rhizosphere persistence (rhizocompetence) [6, 7]. This group serves as an ideal model for studying niche adaptation because of its remarkable genomic plasticity: whereas the core genome encodes essential cellular functions, the highly variable accessory genome contributes to niche-specific ecological interactions [8]. A substantial fraction of this accessory genome is organized into genomic islands (GEIs), discrete DNA regions that are frequently acquired through horizontal gene transfer. By introducing complex genetic modules, GEIs can rapidly expand the ecological and functional capabilities of their hosts. While bacterial rhizosphere adaptation is generally attributed to evolutionary processes, beneficial traits can also arise from horizontal gene transfer (HGT) events, reshaping bacterial genomes and phenotypes [9]. In densely populated and highly competitive environments such as the rhizosphere, HGT represents a rapid mechanism for ecological innovation, enabling bacteria to expand their functional repertoire beyond core genome constraints. Consequently, the acquisition of GEIs via HGT plays an important role in bacterial adaptation within ecological niches colonized by multispecies communities, such as the rhizosphere, by providing access to ecologically relevant gene clusters and functional modules that can mediate niche specialization, interspecies interactions, and host association [10]. Importantly, GEIs are key genomic features contributing to these macro-evolutionary adaptations because complex plant-beneficial and biocontrol phenotypes rely on coordinated multi-gene pathways such as biosynthetic gene clusters or secretion systems, which need to be transferred *en bloc* rather than acquired via stepwise point mutations [11, 12]. For instance, GEI acquisition can convert commensal strains into pathogenic bacteria, as shown for a *Pseudomonas* sp. isolate that incorporated the GEI encoding syringopeptin/syringomycin phytotoxin [12]. Conversely, acquisition of symbiosis-associated GEIs transformed *Mesorhizobium loti,* into a plant symbiont [13]. These examples illustrate how GEIs can act as drivers of ecological transitions, facilitating shifts along the mutualism–pathogenicity continuum and promoting rapid adaptation to plant-associated lifestyles. While the contribution of GEIs in plant pathogenicity has been extensively studied [14], their role in rhizocompetence and plant protection has received comparatively less attention. Analysis of these mobile elements provides a valuable framework for understanding the specialized evolutionary repertoire of plant-beneficial isolates. Among these, *Pseudomonas putida* strain IsoF (IsoF) represents a particularly attractive model because of its well-documented plant-beneficial and biocontrol activities [3].

In this study, we investigated the genomic basis of plant-beneficial traits in IsoF, an effective rhizosphere colonizer originally isolated from tomato roots [15]. IsoF has been reported to trigger ISR and to suppress phytopathogens, suggesting a high degree of ecological competence in the rhizosphere [3, 16–18]. However, the genomic determinants underlying this versatility remain unresolved. To address this, we sequenced and analysed the IsoF genome with a focus on horizontally acquired GEIs. Comparative analyses with closely related *Pseudomonas* PGPR strains were performed to identify lineage-specific traits potentially contributing to rhizosphere adaptation. We further examined selected GEI-encoded functions with predicted roles in interbacterial interactions, biofilm formation, and host-associated fitness. By integrating comparative genomics with functional approaches, we aimed to determine how horizontally acquired gene clusters contribute to rhizosphere fitness and plant-beneficial activity.

## Materials and methods

### Phylogenetic analysis and bioinformatic analysis

For taxonomic classification, the Automated Multi-locus Species Tree (AutoMLST) server was used to identify 42 closely related species to IsoF [19]. From these strains, 85 concatenated MLST sequences were employed in the alignment, employing a maximum likelihood phylogenetic analysis using RaxML GUI 2.0 (ML + RAPID, 100 repetition bootstrap, GTRGAMMA substitution) [20]. For genome-based analysis of *Pseudomonas* groups and GEIs, including the *kib* cluster homologs, the CodonTree service based on Global Protein Families (PGFams) of the Bacterial Genome Tree tool at the Bacterial and Viral Bioinformatics Resource Center (BV-BRC) server was employed to generate the phylogenetic trees [21]. The Type Strain Genome Server (TYGS) was used to confirm the classification. Strains carrying homologs of the investigated GEIs and their corresponding loci were identified using CAGECAT with default parameters [22]. Cluster scores were obtained using CAGECAT, based both on presence and CDS protein sequence homology of elements of the IsoF clusters. In the case of the *kib* cluster, only elements of the T4BSS were included for the analysis. For cyclic lipopeptide (CLP) classification of homologous *pso* clusters, a phylogenetic analysis was performed using MacB protein sequences identified by antiSMASH for each cluster [23], while proteins corresponding to each CLP class were obtained from Girard *et al*. [24]. MacB sequence alignment and tree generation were performed using MUSCLE and NJ-BLOSUM62 default parameters in JalView 2.11.4, respectively [25, 26]. For VirB4/DotO/TraU phylogenetic analysis, sequences were extracted from Needham *et al*. and the IsoF DotO (PisoF_02321) protein sequence was included [27]. Sequences were aligned using MAFFT and phylogenetic reconstruction was performed with FastTree2 [28, 29]. Trees were visualized using the Interactive Tree of Life (iToL) v7 server [30].

Genomic and GEI comparison was performed using Proksee [31], including GEIs predicted using Island Viewer4 and GIPsy [32, 33]. GEIs carrying predicted metabolic, symbiotic, resistance, or metabolic features were indicated. Genomes of selected strains (Table S1) were compared using Proksee BLAST by DNA sequence for closely related *Pseudomonas* strains or by CDS for comparison kib cluster homologs. Conjugative and integrative elements in IsoF genome were determined with ICEfinder [34], while phage regions were investigated using PHASTER [35] and *oriC* location with the Ori-Finder prokaryotic suite [36]. Bacterial systems in the IsoF genome were identified with TxxScan models using MacsyFinder [37, 38]. The VFanalyzer tool of the Virulence Factor DataBase (VFDB) was employed to scan for virulence factors, using *P. aeruginosa* as reference genome [39]. COG analysis was implemented using Genovi [40]. T4BSS effectors within IsoF proteome (Uniprot UP000829686) were screened using DeepSecE [41].

### Bacterial growth conditions and media

Bacterial strains employed in this study are detailed in Table S2. Overnight cultures were grown in Lysogeny Broth Lennox (LB, Difco, 240210) at 37°C (*Escherichia coli* species) or at 30°C (*Pseudomonas* species). Experiments were performed in LB, LB media without salt (LB-; 10 g L^−1^ Tryptone, 5 g L^−1^ yeast extract, Difco 211921 and 210929), or AB medium supplemented with sodium citrate 10 mM (ABC) [42], as indicated. When indicated, ABC was supplemented with 0.2% casamino acids (ABCAS). *Pseudomonas* isolation agar (PIA, Difco, 292710) was employed for the selection of *Pseudomonas* mutants and transconjugants. When appropriate, media were supplemented with antibiotics at the following final concentrations: for *Pseudomonas* species, 20 μg ml^−1^ gentamicin (Gm) and 20 μg ml^−1^ tetracycline (Tet), and for *E. coli*, 100 μg ml^−1^ ampicillin (Amp), 10 μg ml^−1^ Gm, 25 μg ml^−1^ kanamycin (Km), and 10 μg ml^−1^ Tet.

### Generation of IsoF deletion mutants

Defined IsoF derivatives carrying single (ΔpsoABC, Δppu, ΔT4B), double (ΔpsoABC ΔT4B), and triple (Δppu ΔpsoABC ΔT4B) gene deletions of target GEIs were generated using a SceI-based mutagenesis system, as previously described [3, 43]. Briefly, two homology sequences flanking the region to be deleted were cloned into the suicide vector pGPI-SceI::TetAR and introduced into *E. coli* S17–1λpir cells. Plasmids were transferred into IsoF via tri-parental mating, using *E. coli* DH5α pRK2013 helper strain. Overnight cultures of the strains were washed with 0.9% NaCl and mixed in a 2:2:1 ratio (helper:donor:recipient). The mixture was spotted on LB plates in 50 μl drops aliquots and incubated overnight at 30°C. Resulting bacteria spots were resuspended in 1 ml of 0.9% NaCl, plated on PIA Tet, and incubated overnight at 30°C. To generate the cleavage in the SceI site, the pDAI plasmid was introduced in IsoF via tri-parental mating using the pRK2013 helper plasmid. PIA Gm and Gm-Tet plates were used to select sensitive exconjugants to Tet, which were screened by PCR using the corresponding check primers. PIA and PIA Gm plates were used to select deletion mutants lacking the pDAI plasmid. The deletion mutants were verified by sequencing on the flanking regions. See Table S3 for employed primers and restrictions enzymes.

### Generation of fluorescently tagged strains

A mini-Tn7 system was used to integrate the locus encoding a red fluorescent protein (mCherry) into the genome of the generated deletion mutants listed in Table S2 [44]. Chromosomally tagged strains were generated by tri-parental mating, using *E. coli* S17–1 λpir strains harbouring the plasmid pUCT18-mini-Tn7 or the Tn7 helper plasmid pUX-BF13, as previously described [3, 44–46]. Fluorescent colonies were selected on PIA Gm plates.

### Biofilm formation assay

Biofilm formation was quantified as described by Huber *et al*. [46]*.* Overnight bacterial cultures in ABC were washed and normalized with 0.9% NaCl to an optical density at 600 nm (OD_600_) of 0.01. A 96-well sterile polystyrene microtitre plate was inoculated with 100 μl of ABC medium and 1 μl of normalized culture per well, and the plate was incubated for 48 h at 30°C. Absorbance at 550 nm was measured with a Synergy H1 microplate reader (BioTek instruments) and medium was carefully discarded. Staining was carried using 100 μl of crystal violet per well, and plates were incubated for 30 min at room temperature (RT). Solutions were discarded, and each well was washed twice with 100 μl ddH_2_0. Crystal violet was resolved out of the biofilm by incubating the plate with 120 μl of dimethyl sulfoxide (DMSO) per well for 20 min at RT. Absorbance at 570 nm was quantified and biofilm index (BI) was calculated as a percentage using the 570:550 nm signal ratio, as a normalization by bacterial growth to compare biofilm amounts among samples.

### Analysis of bacterial growth and morphology

Growth curves of IsoF mCherry-tagged deletion mutants were performed in LB, LB-, and ABC cultures. Briefly, overnight LB- cultures were washed and normalized to an OD_600_ of 0.01 in 0.9% NaCl. Aliquots of 100 μl were inoculated into a sterile 96-well sterile polystyrene microtiter plate per well and incubated in a Synergy H1 microplate reader (BioTek instruments) with orbital agitation at 30°C for 32 h. Absorbance at 600 nm was measured every 20 min and analysed using GraphPad Prism v8.4.1.

Bacterial morphology was examined under a confocal laser scanning microscope (CLSM; Leica TCS SPE, DM5500) with a ×100/1.44 oil immersion objective. Adhesive silicon isolators (Grace BioLabs, JTR8R-1.0; 8–9 mm Ø × 1 mm depth) were filled with ABC medium containing 1% agar and inoculated with 1 μl of culture normalized to an OD_600_ of 0.01. Pictures were taken after 16-h incubation at RT and analysed using ImageJ [47].

### Contact-dependent killing assay

Contact-dependent killing (CDK) assays in mixed microcolonies were performed similarly to previous studies [3]. Briefly, overnight LB liquid cultures were adjusted to an OD_600_ of 1 in 0.9% NaCl and Colony-forming units (CFUs) of each competitor were determined by plating bacterial dilutions. Competitors were mixed in a 1:1 ratio, and ABCAS plates were inoculated with 5 μl of mixed cultures. Pictures of colonies were taken after 24 h using a Leica M205 FCA Stereo Microscope. Two mixed macrocolonies were recovered and resuspended in 500 μl of 0.9% NaCl. Serial dilutions in 0.9% NaCl were prepared and plated on PIA and PIA Gm. Plates were incubated at 30°C for 24 h, and CFUs were counted.

To investigate the killing range of IsoF, a CDK monolayer assay was performed using a CLSM (Leica TCS SPE, DM5500) as described in previous studies [3]. Adhesive silicon isolators (Grace BioLabs, JTR8R-1.0; 8–9 mm Ø × 1 mm depth) containing 1% agar and supplemented with 4 μg ml^−1^ propidium iodide (PI, ThermoFisher, P3566) were inoculated with 1 μl of a 1:1 mixed bacterial culture (IsoF::GFP and competitor strain). Killing was monitored using a CLSM (Leica TCS SPE, DM5500). Pictures were acquired after 16-h incubation at RT. Image analysis was conducted using ImageJ [47].

### Drop collapse assay

Biosurfactant activity of putisolvin was analysed using a modified version of the previously described drop collapse assay [17]. Briefly, mutants in the clusters of interest were grown in ABC medium for 24 h and 1 ml of each culture was pelleted. Recovered supernatants were filtered, and 100 μl droplets on parafilm were observed after 5 min for drop shape.

### Bacterial motility assay

Overnight LB-liquid cultures were washed and adjusted with 0.9% NaCl to an OD_600_ of 0.01. Motility assays were performed with modified versions of previously described protocols [48, 49]. For swarming motility assays, 1 μl of normalized culture was placed on top of ABC 0.4% agar plates and dried for 30 min. For swimming motility, ABC 0.3% agar plates were slightly pierced with a pipette tip while depositing 1 μl of the adjusted culture. Plates were incubated at 30°C for 120 h, and pictures were taken every 24 h.

### Antifungal activity

Antifungal activity against *Alternaria alternata* was investigated as previously described [50]. Briefly, 20 μl of overnight cultures were spotted at three positions on malt agar (DIFCO) 2% agar plates and incubated 24 h at 30°C. A fungal inoculum (5 mm Ø) from the outer (youngest) edge of the fungal mycelium was placed onto the centre of the plate, and plates were incubated at RT in the dark. Inhibition zones were recorded after 4 days. Percentage of inhibition was calculated as the ratio inhibition:total area of the plate and normalized to the 100% inhibition reference set for IsoF wild type.

### Rhizosphere persistence assay

The persistence of mCherry-tagged deletion mutants of IsoF in the rhizosphere of *Solanum lycopersicum* cv. MicroTom tomato plants was tested following a modified plant-associated bacteria recovering protocol [3]. Tomato seeds were surface-sterilized by treatment with 1% sodium hypochlorite for 10 min and rinsed with sterile deionized water. Seeds were stratified at 4°C in the dark for 2 days and subsequently sowed on 0.8% water agar plates and kept at 30°C in the dark for 2 days. Seedlings were incubated in a light chamber at 22°C in long-day photoperiod conditions (16 h, 100 μmol m^−2^ s^−1^ photon flux, 18°C in dark regime, 60% relative humidity) for 7 days until lateral root emergence. Prior to transplantation, nonsterile soil (in 7 × 7 × 6 cm pots, Desch Plantpak, 100003) was treated with 50 ml of bacterial suspensions of the generated deletion mutants, adjusted at an OD_600_ of 1 in liquid LB-. Plants were grown in long-day photoperiod conditions and bacteria were recovered after 21 days from rhizosphere and soil fractions by incubating manually cleaned roots and distant-to-root soil samples, respectively, in shaking conditions (200 rpm) for 20 min followed by a 50 Hz sonication water bath (Branson 2210 R-MT, Germany) at 22°C for 20 min in Falcon tubes filled with 2.5 ml of 0.9% NaCl. Recovered cell suspensions were diluted and plated on PIA Gm, incubated at 30°C overnight, and CFUs were normalized to fraction weight. Due to differences in plant survival, the number of seedlings varied among treatments and replicates. Each treatment included between 22 and 24 plants in total across four independent biological replicates. Exact numbers are provided in Table S4.

### Statistics and reproducibility

Statistical significance was evaluated using appropriate tests as specified in the corresponding figure legends. Analyses were conducted using GraphPad Prism v8.4.1. A threshold of *P* < .05 was considered significant. Student’s *t-*test and analysis of variance (ANOVA) with Dunnet’s comparison were employed as indicated in figure legends, with significance levels denoted by asterisks: ^*^P < .05, ^**^P < .01, ^***^P < .001, ^****^P < .0001. No statistical method was used to predetermine sample size. The experiments were not randomized, and investigators were not blinded to group allocation or outcome assessment. No data were excluded from the analyses.

## Results

### IsoF is part of the *Pseudomonas capeferrum* group

**Figure 1:**
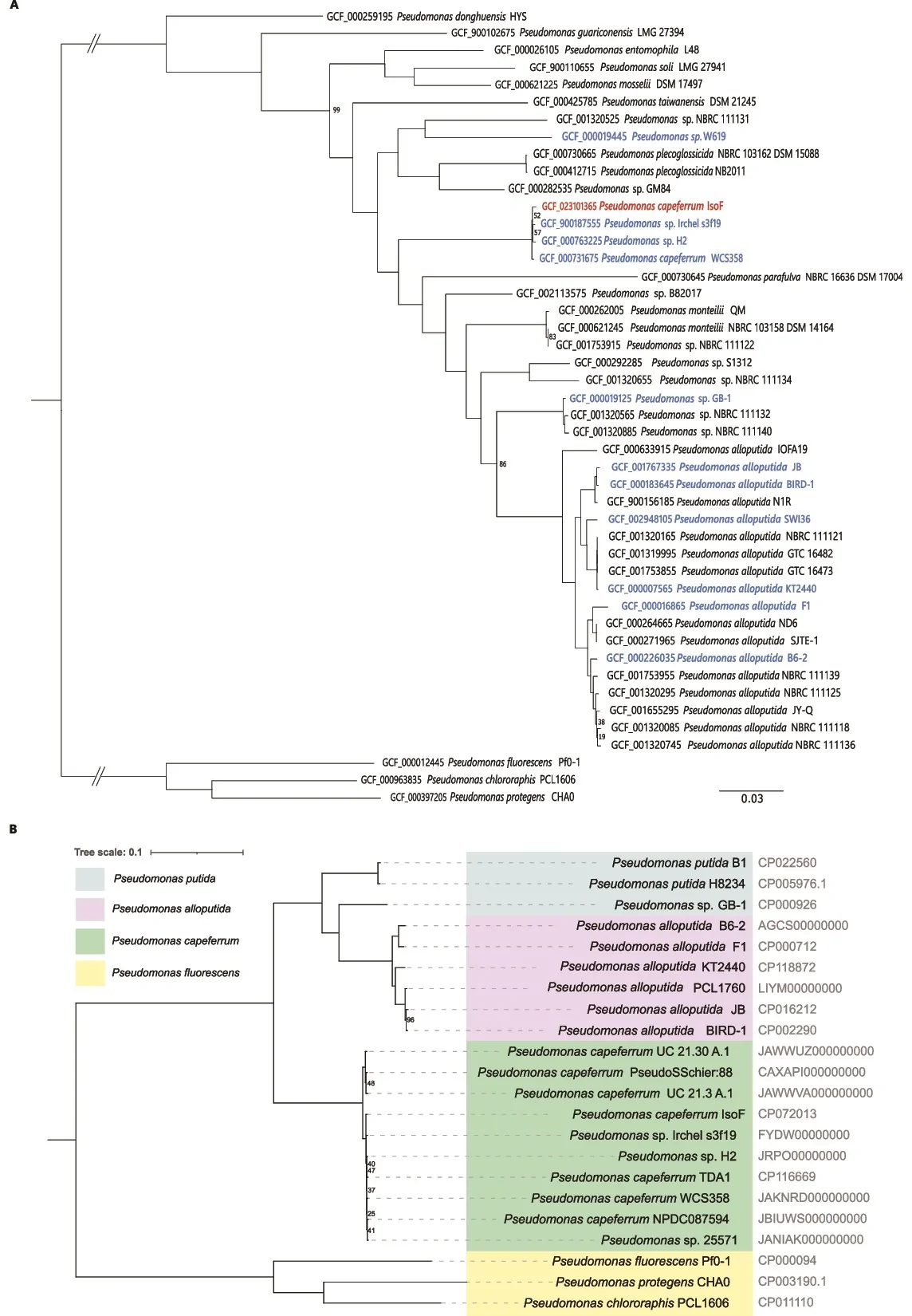
Phylogenetic analysis of the IsoF genome. (A) Maximum likelihood phylogenetic analysis based on 85 concatenated MLST sequences from 46 selected *Pseudomonas* strains. IsoF is highlighted in red, while other genomes analysed within this study are shown in blue. (B) Phylogenetic tree based on BV-BRC Global Protein Families (PGFams) using members of the *P. alloputida* and *P. capeferrum* families [21, 108]. The *P. fluorescens* subgroup served as outgroup. Nodes with a bootstrap score lower than 100 are indicated. *Pseudomonas alloputida* classification was based on Passarelli-Araujo *et al*. [101].

The IsoF genome (NCBI accession number NZ_CP072013) was assembled as a single chromosome of 5 929 333 bp and an average guanine-cytosine (GC) content of 62.56% (coverage 120×). Dot plot analysis did not show overlapping ends in the single-contig assembly, and thus, no end trimming was required (Fig. S1). Automatic annotation revealed 22 rRNAs, 77 tRNAs, 1 tmRNA, and 5242 putative protein-coding sequences (CDSs), including 852 genes encoding hypothetical proteins (16.4% of total CDSs). Based on 16S rDNA sequence analysis and phenotypic characteristics, IsoF was initially classified as a *P. putida* [3, 15, 17, 51]. To refine its taxonomic assignment, we conducted a phylogenetic analysis using concatenated sequences from 85 single genes from 46 closely related *Pseudomonas* strains (Fig. 1A) [19]. The resulting tree placed IsoF within the *P. capeferrum* WCS358 branch, distinct from other *P. putida* strains. Complementary TYGS genome-based taxonomic classification and PGFam-based phylogenetic analyses with members of the *P. capeferrum* subgroup and related *P. (allo)putida* strains confirmed the placement of IsoF within the *P. capeferrum* lineage, alongside the unclassified *Pseudomonas* sp. H2, *Pseudomonas* sp. 25571, and the closely related strain *Pseudomonas* sp. Irchel sf319 (Fig. 1B; Table S5). Consequently, we refer to the strain as *P. capeferrum* IsoF (IsoF) hereafter.

### *P. capeferrum* IsoF possesses a diverse array of unique GEIs

Previous studies have shown that IsoF can promote ISR against phytopathogens and efficiently colonize and protect the tomato rhizosphere, suggesting its potential application as a biocontrol strain [3, 15, 18, 51]. Given the ecological versatility of closely related *Pseudomonas* species, we hypothesized that horizontally acquired GEIs contribute to the beneficial traits of IsoF [3, 52, 53]. We therefore examined whether GEIs encode functions linked to rhizosphere fitness, bioremediation, and biocontrol. We identified 40 putative GEIs representing 15.1% of the IsoF genome (Table S6). These regions were unevenly distributed across the chromosome and displayed hallmarks of horizontal acquisition, based on discrepancies in GC content between GEIs and the core genome [10].

**Figure 2:**
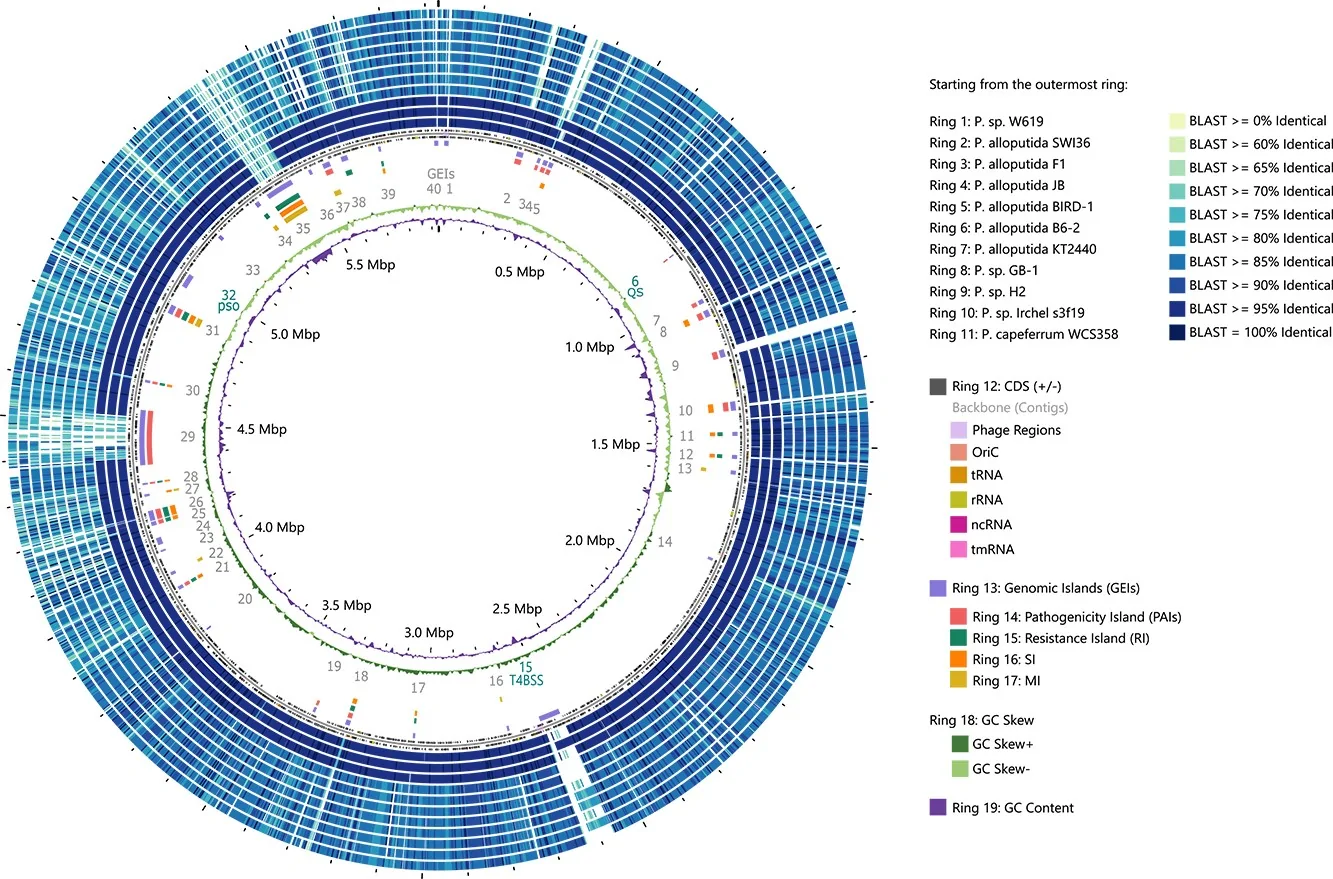
Genome comparison analysis. Circular whole-genome comparison indicating predicted genomic islands (GEIs) in *P. capeferrum* IsoF. Each ring corresponds to a sequence alignment using the IsoF genome as reference. GEIs were predicted using GIPSy, ICEfinder, and IslandViewer4, while prophage regions were identified using PHASTER. OriFinder was employed to identify the origin of replication (oriC). The circular comparative analysis was generated with Proksee. Predicted GEIs include metabolic island (MI), pathogenicity island (PAI), resistance island (RI), and symbiotic island (SI).

A comparative genomic analysis between the IsoF genome and selected closely related *P. (allo)putida* and *P. capeferrum* strains (Table S1) revealed multiple regions lacking sequence homology, consistent with a distinct GEI repertoire in IsoF (Fig. 2). To systematically assess these regions, we integrated predictions from complementary *in silico* tools: (i) IslandViewer4, to predict GEIs in IsoF genome; (ii) GIPSy, which detects four different classes of GEIs; and (iii) the ICEfinder server, which identifies integrative and conjugative elements (ICEs) in bacterial genomes [32–34]. Several of the predicted islands were classified as miscellaneous GEIs, potentially involved with antimicrobial resistance, metabolism, pathogenicity, or symbiosis (Fig. 2; Table S6). Together, these analyses indicate that IsoF harbours a substantial and functionally diverse accessory genome, consistent with recent horizontal acquisition events.

Furthermore, we identified two incomplete prophage regions in the IsoF genome using PHASTER [35], one within GEI 1 and the other adjacent to GEI 15 (Fig. 2; Table S7). Several GEIs also contained genes associated with horizontal mobility, including integrases, recombinases, and transposases highlighting their potential for genomic plasticity and ecological adaptability. Moreover, tRNA-encoding genes were identified flanking 10 of the predicted GEIs, consistent with their previously described role as frequent insertion sites for horizontally acquired elements (Table S6) [10, 54].

### Functional diversity of IsoF GEIs

The diverse functions encoded within the IsoF GEIs are reflected by the broad spectrum of clusters of orthologous groups (COGs) represented within them (Fig. S2). Several of the predicted GEIs encode elements contributing to adaptability, growth, and persistence in the rhizosphere (Table S8). For example, genes related to cadmium, cobalt, and zinc transport systems, as well as to arsenic resistance, were detected within GEIs, suggesting tolerance to heavy metals (GEIs 14, 21). Other GEIs carried genes involved in the uptake of putrescine and sulfonate, both important micro-nutrients in the rhizosphere (GEI 11, 13, 25) [2, 55, 56]. In addition, various GEIs encode elements related to antimicrobial resistance (GEIs 5, 11, 17, 26, 38), including regulators and proteins involved in β-lactam, colistin, and aminoglycoside tolerance (Table S6). Despite this genetic potential, IsoF remained sensitive to colistin and aminoglycoside antibiotics, indicating differential expression or regulation under laboratory conditions. Regarding bioremediation, genes associated with plastic degradation and putative sarcosine oxidase–encoding genes linked to glyphosate bioremediation were identified (GEI 22, 26, 28; Table S9) [57].

Closer inspection of the predicted pathogenicity islands (PAIs) suggested that these regions may contribute to adaptability, competitiveness, and general fitness rather than to virulence (Table S6). This interpretation is based on homology with elements involved in these processes in other species and is consistent with the limited number of predicted virulence factors identified in the IsoF genome (Table S10). For example, genes encoding pilin and fimbriae subunits found within some PAIs are typically linked to adhesion and surface attachment, key traits for bacterial colonization, that may enable IsoF to adhere to plant roots (GEIs 2, 8, 18) [58]. Other GEI-encoded genes may participate in iron acquisition in IsoF, such as receptors for the siderophore pyoverdine (GEIs 8, 12) [59]. Siderophores not only provide access to essential iron but also mediate competitive suppression of co-occurring microorganisms [60]. Peptidoglycan (PG) O-acetylation by the O-acetyltransferase OatA (GEIs 4, 39, 40) protects the PG from lytic transglycosylases and lysozyme-mediated degradation. Regulation of *oatA* expression in IsoF may thus support multiple ecological functions, including autolysis prevention, immune evasion, and facilitation of secretion system assembly [61, 62]. Restriction-modification (RM) system elements were also found in IsoF GEIs, potentially contributing to defence against foreign DNA (GEIs 10, 15) [3, 63].

Additional elements found in the predicted GEIs included putative toxins, such as colicin and pyocin bacteriocin–related genes, which inhibit the growth of susceptible bacterial strains (GEIs 1, 37) [64]. Among the identified effector proteins, amidases, rearrangement hotspot (Rhs) proteins, and glycosyltransferases are known to antagonize competitors after contact-dependent delivery into neighbouring cells (GEIs 2, 5, 11, 25) [65]. Notably, an interbacterial killing T4BSS (GEI 15) [3], a type VI secretion system (T6SS), and other macromolecular bacterial systems were identified in the IsoF genome using TXSScan (Table S11) [37, 38].

Collectively, these GEIs likely enhance IsoF fitness in the rhizosphere by promoting competition, colonization, and resource acquisition. To assess whether specific islands contribute to this fitness, we focused on three GEIs likely involved in rhizosphere persistence and colonization: the *pso* putisolvin biosynthetic cluster (GEI 32), the *ppu* quorum sensing (QS) system (GEI 6), and the T4BSS encoded within the *kib* cluster (GEI 15).

### The unique T4BSS of IsoF is encoded by GEIs

**Figure 3:**
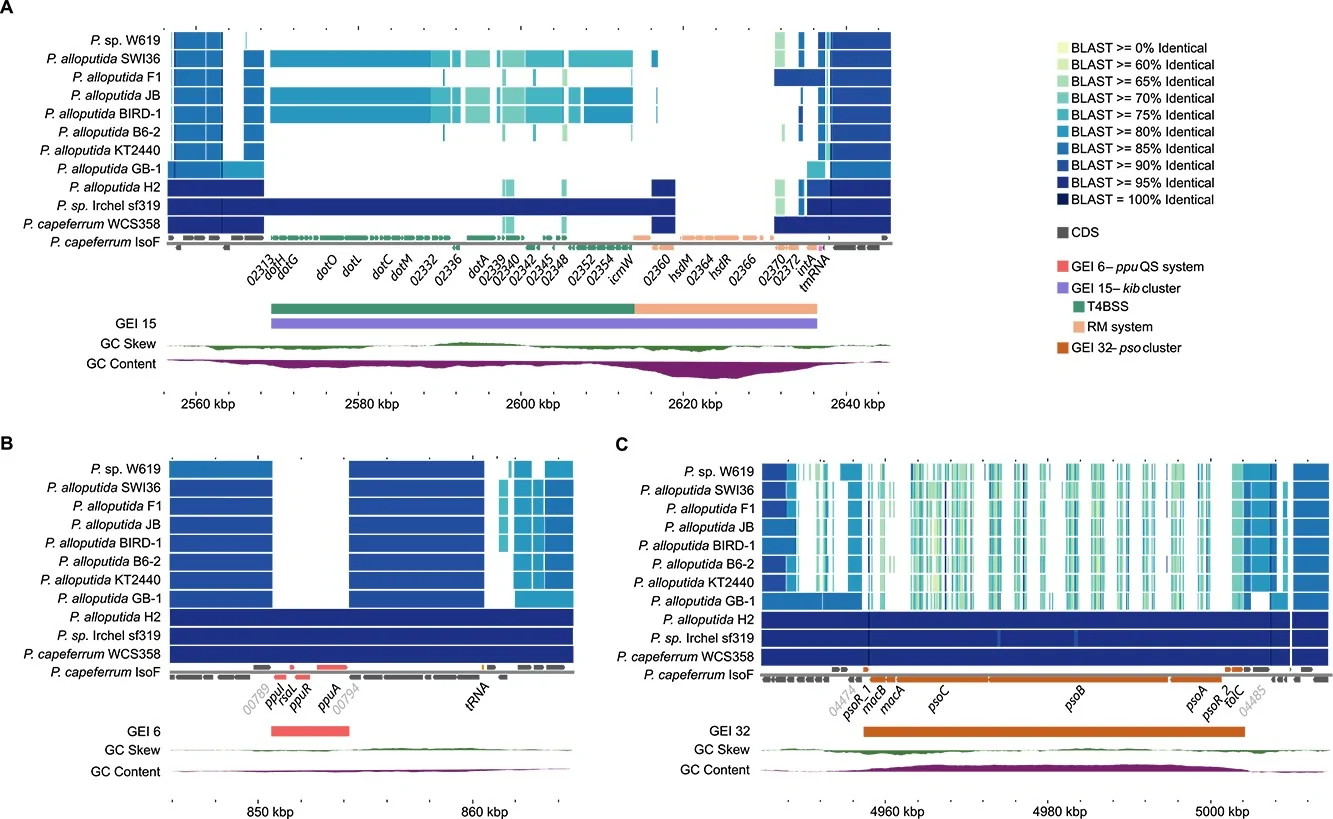
Sequence alignment comparison with IsoF GEIs. Genome alignment of the selected Pseudomonas strains and the GEI regions corresponding to the *kib* cluster (A, GEI 15), the *ppu* QS system (B, GEI 6), and the *pso* cluster (C, GEI 32) of IsoF. Analysis was performed using Proksee [31].

Bacterial secretion systems are frequently located in mobile islands, such as the T3SS of *P. syringae*, within the *hrc* and *hrp* PAIs; the T6SS of *P. aeruginosa*, as part of the *hcp* secretion island; and the T6SS of *E. coli*, *Salmonella typhimurium,* and *Vibrio cholerae* [66–70]. The interbacterial killing T4BSS of IsoF is encoded within GEI15, alongside an RM system and an integrase at one of its ends, adjacent to a tmRNA element, commonly associated with GEIs acquired via HGT (Fig. 3A) [10, 54]. A phylogenetic analysis based on VirB4/DotO/TraU protein sequence homology (Fig. S3) placed the IsoF T4BSS in the type IV protein secretion systems of the mating-pair formation type I subgroup (pT4SSi), consistent with its role as an effector delivery T4BSS [3, 71, 72]. Within this clade, the IsoF T4BSS clusters most closely with systems found in the clinical isolates *P. putida* H8234 and *Yersinia pseudotuberculosis* IP31758 (Fig. S3) [3, 73].

**Figure 4:**
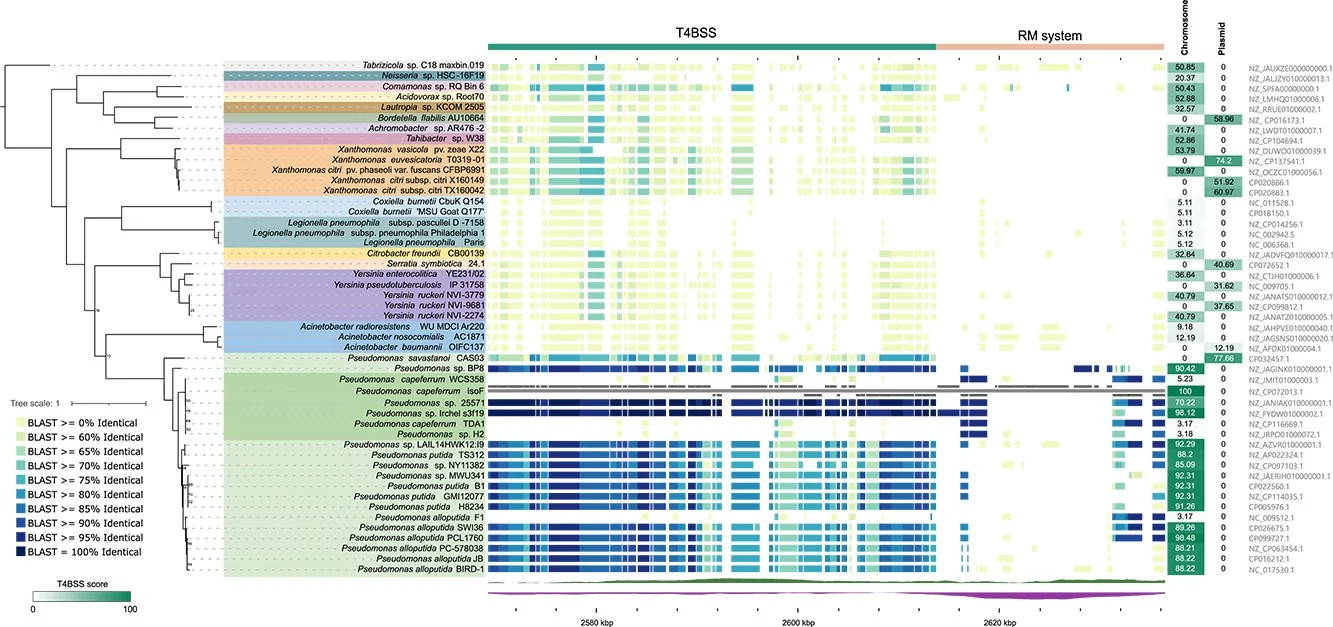
Distribution of elements showing homology to the IsoF *kib* cluster. IsoF GEI 15 comparison with bacterial species carrying homologs of the IsoF T4BSS. The T4BSS score, which is based both on presence and CDS protein sequence homology of elements of the T4BSS cluster using the IsoF genome as reference, is indicated for clusters identified either within chromosomes or plasmids. CDS numbers indicate genomic locus (*PisoF_023xx*). Analysis was performed using BV-BRC, CAGECAT, and Proksee [21, 22, 31].

To assess the ecological distribution of IsoF T4BSS homologs across different genera, strains carrying T4BSS *loci* were identified and scored using the CAGECAT web server, followed by genomic comparison and phylogenetic analysis using Proksee and BV-BRC tools, respectively [21, 22, 31]. Homologs of the IsoF T4BSS cluster were detected across diverse phylogenetic groups, including Gammaproteobacteria (*Acinetobacter*, *Citrobacter*, *Pseudomonas*, *Xanthomonas*, *Yersinia*), Betaproteobacteria (*Bordetella*, *Acidovorax*, *Comamonas*), and Betaproteobacteria/Neisseriales (*Neisseria*), among others (Fig. 4). The closest homologous clusters were generally found in *Pseudomonas* genomes, although plasmid-borne systems were also observed. Notably, the closest non-*Pseudomonas* homolog was identified on plasmid p3191.2 of *Xanthomonas euvesicatoria* T0319-01 (NCBI entry NZ_CP137541), which is consistent with a possible plasmid-associated origin and HGT of the IsoF T4BSS. Some *Pseudomonas* strains carried only partial T4BSS loci, which may encode effector–immunity protein (E-I) pairs or orphan immunity proteins, potentially conferring resistance to the IsoF T4BSS. This is analogous to orphan immunity islands described for T6SSs [74, 75].

To identify potential antibacterial effectors of the IsoF system, T4SS effector candidates were predicted by querying the IsoF proteome against DeepSecE [41]. This analysis identified three putative T4SS effectors within the *kib* cluster: PisoF_02332, a proposed immunity protein carrying the FxxxLxxK translocation signal recognized by the *Legionella* LvgA protein [76]; PisoF_02340, containing a DUF2335 domain similar to the type VI lipase effector (Tle1) of T6SSs [77]; and PisoF_02350, of unknown function (Table S12). These proteins show low to no homology with T4BSSs of human pathogens, suggesting that they may represent system-specific factors potentially involved interbacterial antagonism.

### Fitness adaptation driven by IsoF GEIs

**Figure 5:**
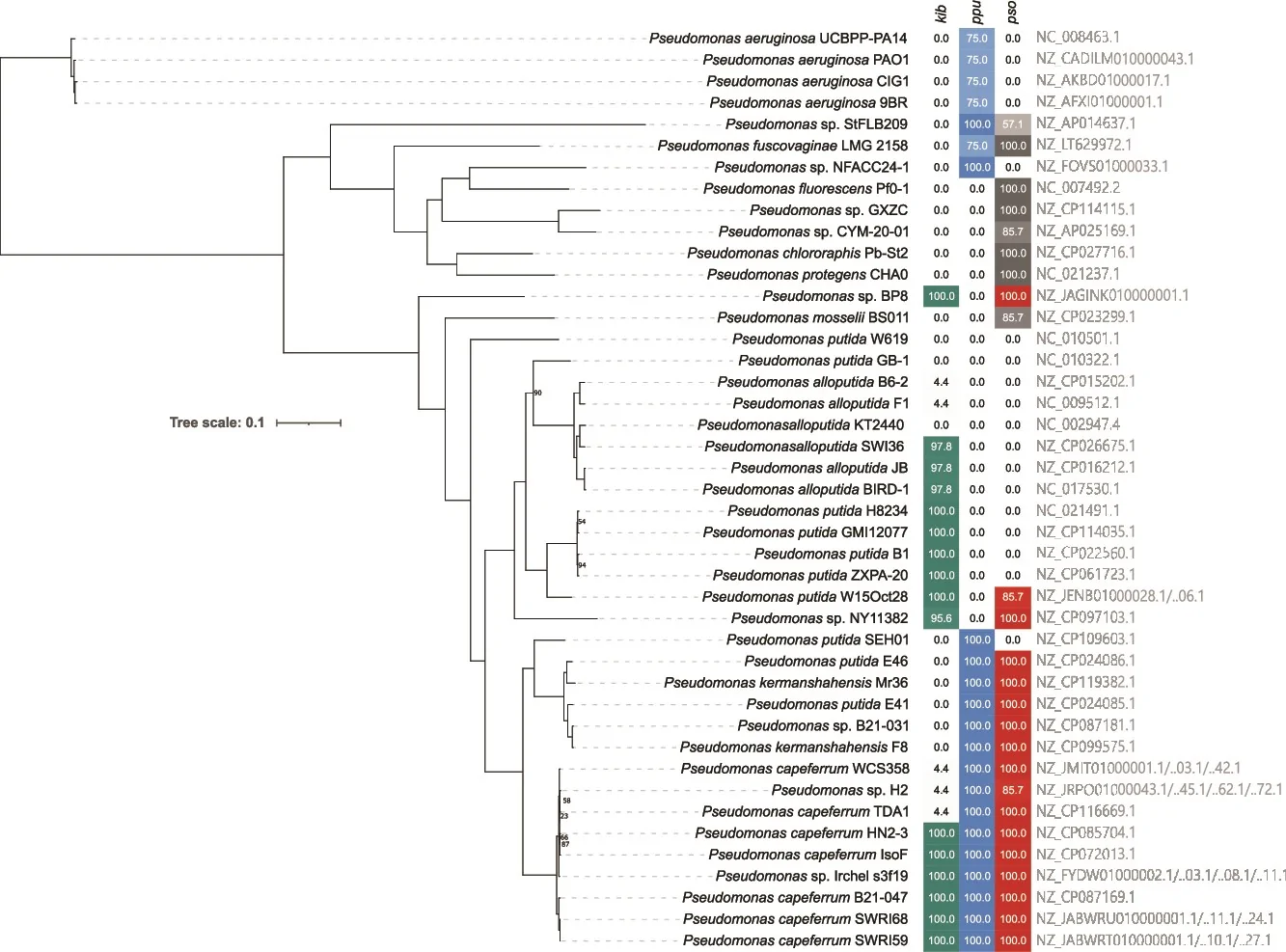
Co-presence analysis of IsoF GEIs. Analysis of the co-occurrence of the T4BSS-*kib* (GEI 15), the *ppu* (GEI 6), and the *pso* (GEI 32) clusters within the genome of *Pseudomonas* strains. Homologous *pso* clusters encoding for the production of other cyclic lipopeptides are indicated in grey. Cluster scores are based both on presence and CDS protein sequence homology of elements of each cluster, using IsoF genome as reference.

QS systems in PGPRs are known to regulate a variety of plant-beneficial properties in a cell-density-dependent manner [78, 79]. In IsoF, the *ppu* QS system (GEI 6) regulates the *pso* cluster (GEI 32), involved in the biosynthesis of putisolvin, a cyclic lipopeptide (CLP) implicated in swarming motility, biofilm formation, and potential biocontrol activity [3, 17, 51, 80]. Comparative analysis of these islands across different selected strains using IsoF as reference revealed close homology with strains closely related to IsoF (Fig. 3). The difference in GC content between the *ppu* (60.6%, GEI 6) and the *pso* (66.9%, GEI 32) GEIs (Fig. 3; Table S6), combined with the presence of strains carrying only one of these GEIs (Fig. 5), suggest that the two islands were acquired through independent HGT events. Despite their independent origins, these clusters have functionally co-evolved, with the *ppu* QS system regulating the *pso* gene cluster, thereby influencing swarming motility, removal of dead competitor cells, and biofilm formation in IsoF [3, 17]. Interestingly, among the different strains carrying homologous CLP clusters (Fig. S4), only three strains lacking the *ppu* QS system while retaining the *pso* biosynthetic cluster were identified, raising questions about alternative regulatory mechanisms controlling putisolvin production in these isolates (Fig. 5).

The previously reported biocontrol capabilities of IsoF via its T4BSS, combined with the unique properties of the *ppu* and *pso* clusters in its genome, suggest that some of these horizontally acquired GEIs may contribute to the strong rhizosphere colonization and adaptation capabilities of IsoF. This specific combination of clusters (*ppu*, *pso,* and *kib*) constitutes a distinct GEI repertoire, observed only in six strains of the *P. capeferrum* group through comparative genomics across the entire NCBI database: *P. capeferrum* SWRI59 [81], *P. capeferrum* SWRI68 [81], *Pseudomonas* sp. Irchel s3f19 [82], *P. capeferrum* B21-047 [83], and *P. capeferrum* HN2-3 [80] (Figs 3A and 5, Fig. S5).

### Functional validation of IsoF GEIs in biofilm formation and rhizosphere fitness

**Figure 6:**
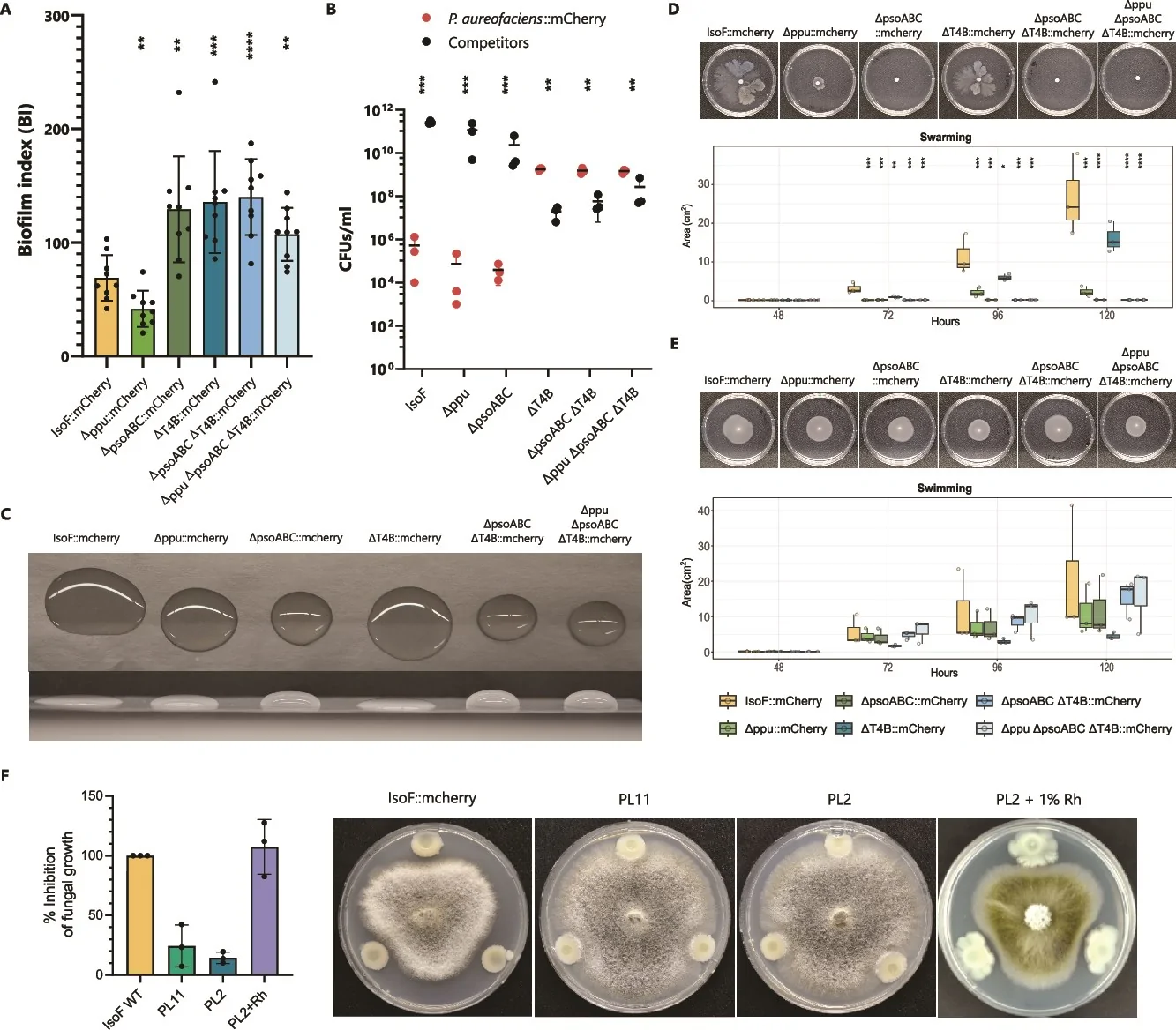
Phenotypic characterization of GEI mutants. (A) Crystal violet assay for the analysis of biofilm production. (B) Contact-dependent killing (CDK) assay of selected mutants against *P. aureofaciens* tagged with mCherry (*P. aureofaciens*::mCherry). (C) Surface tension was tested through drop collapse assay using filtered supernatant fractions of IsoF deletion mutants, indicating the presence of putisolvin. (D) Bacterial swarming motility assay. (E) Bacterial swimming motility assay. (F) IsoF *psoA* knockout (PL11) and conditional (PL2) mutants were tested in antifungal assays against *A. alternata*. Inhibition zones were recorded after four days, calculated as the percentage of the inhibition area divided by the total plate area, and normalized to IsoF wild type. Expression of *psoA* in the PL2 conditional mutant was induced with 1% rhamnose (Rh). Fluorescently tagged (mCherry) and non-tagged deletion mutants of the *ppu* (Δppu), *pso* (ΔpsoABC), and *kib* (ΔT4B) clusters were employed for the rest of experiments. Data are mean ± s.d.; significance was assessed using an unpaired *t-*test for bacterial CDK assay and an analysis of variance (ANOVA) with Dunnett’s multiple comparison for area of bacterial motility using IsoF::mCherry as reference; ^*^P < .05; ^**^P < .01; ^***^P < .001; ^****^P < .0001.

To test the contribution of the *ppu*, *pso,* and *kib* clusters to IsoF fitness, single and combination deletion mutants (ΔpsoABC, Δppu, ΔT4B, ΔpsoABC ΔT4B, Δppu ΔpsoABC ΔT4B) were generated and phenotypically characterized. All mutants consist of clean, markerless deletions of entire genomic islands spanning ~10–65 kb (Table S6) and encompassing multiple operons and regulatory elements, rather than single-gene knockouts. These mutants showed no differences in growth rate in liquid culture or in cell morphology compared to IsoF wild type (Fig. S6). Crystal violet assays revealed an increase in biofilm production in the corresponding T4BSS and *pso* mutants (Fig. 6A). The increased biofilm in ΔpsoABC mutants likely reflects the absence of putisolvin, a biosurfactant involved in biofilm dispersal, whereas the mechanism in T4BSS mutants remains unclear. Notably, the *ppu* QS mutant produced less biofilm than the wild type, whereas deletion of the QS-regulated *pso gene* cluster increased biofilm formation, suggesting that additional, yet unidentified, QS-regulated factors influence biofilm development. In competition assays, only the T4BSS mutants were impaired, whereas deletion of neither the *ppu* nor the *pso* gene cluster affected competitive fitness (Fig. 6B), indicating that the IsoF T4BSS is not regulated by the *ppu* QS system. Consistent with previous studies, mutants in the *ppu* and *pso* clusters were impaired in putisolvin production (Fig. 6C) and consequently exhibited reduced swarming motility (Fig. 6D) [17], while swimming motility remained unaffected (Fig. 6E).

**Figure 7:**
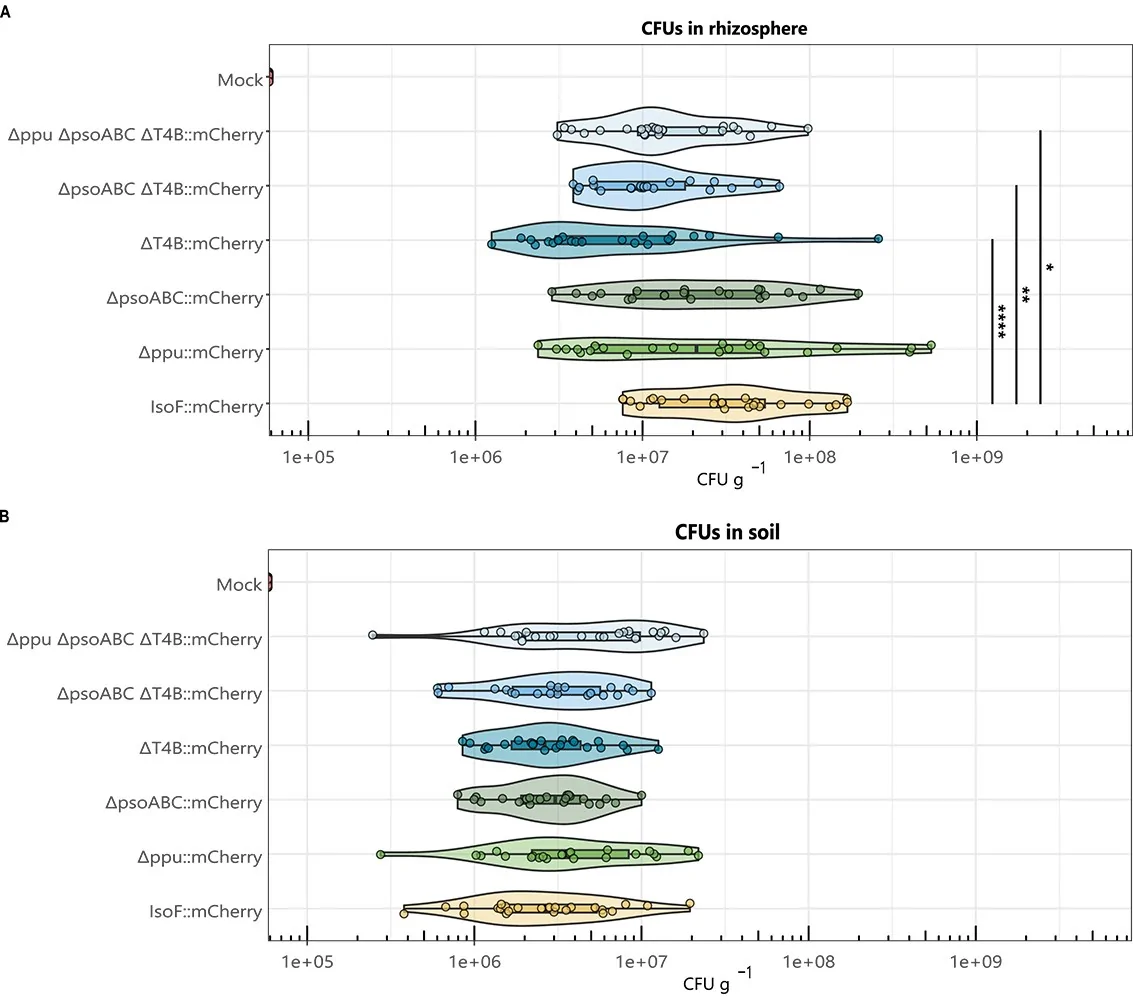
Rhizosphere persistence of deletion mutants in IsoF GEIs. Recovered CFUs from rhizosphere (A) and soil (B) fractions, normalized by fraction weight. Fluorescently tagged (mCherry) deletion mutants of the *ppu* (Δppu), *pso* (ΔpsoABC), and *kib* (ΔT4B) clusters were employed. An analysis of variance (ANOVA) with Dunnett’s multiple comparison was performed using IsoF::mCherry as reference; ^*^P < .05; ^**^P < .01; ^***^P < .001; ^****^P < .0001.

To evaluate the role of these GEIs under ecologically relevant conditions, the soil-rhizosphere fitness of these deletion mutants was assessed by inoculating normalized liquid cultures into nonsterile substrate of 7-day-old tomato seedlings. CFUs were recovered from both distant (soil) and adjacent (rhizosphere) fractions associated with the roots after 28 days. T4BSS mutants exhibited reduced persistence in the rhizosphere compared to the wild type (Fig. 7). This reduction correlates with the impairment in contact-dependent killing (Fig. 6B), as this system exhibits a wide range of activity against all tested Gram-negative bacteria to date (Fig. S7) [3]. Increased biofilm formation alone is unlikely to explain the reduced rhizosphere persistence of the T4BSS mutants, as the ΔpsoABC mutant displayed a similar biofilm phenotype without exhibiting a persistence defect.

In contrast, mutants of the *pso* and *ppu* clusters showed no significant differences in rhizosphere persistence relative to the wild type, indicating that swarming motility and putisolvin production are not essential for IsoF survival in this niche under the conditions tested. Similarly, recovered CFUs from bulk soil were lower than from rhizosphere fractions, with no significant differences among mutants and wild type. These results suggest that IsoF fitness and T4BSS activity are likely influenced by root exudates or other rhizosphere-specific factors, highlighting the ecological importance of this secretion system for competitive persistence (Fig. 7).

Putisolvin has also been reported to exert zoosporicidal and antifungal activities through diffusion [84, 85]. To assess these activities in IsoF, an antifungal assay was performed using the previously generated *psoA* deletion mutant PL11 and its rhamnose-inducible conditional derivative PL2 [17]. Our results confirm a putisolvin-dependent antifungal activity in IsoF (Fig. 6F), consistent with the antimicrobial role of CLPs in many *Pseudomonas* species [85, 86]. This activity may protect IsoF against fungal antagonists and contribute to the protection of the plant host from fungal phytopathogens in natural environments.

## Discussion

The *P. capeferrum* lineage comprises naturally occurring environmental PGPR isolates with strong rhizosphere competence. For example, *P. capeferrum* strain WCS358, originally isolated from the potato rhizosphere, suppresses plant diseases through pyoverdine (PVD358) siderophore production and iron sequestration, inspiring the lineage name *capeferrum* (‘seizes iron’) [87–89]. Similarly, *P. capeferrum* CFPB2461 exhibits beneficial properties in bean roots [90], while *P. capeferrum* F8 modulates the production of secondary metabolites by the biocontrol agent *P. protegens*, such as the antimicrobial pyoluteorin [91]. In WCS358, gluconic and 2-keto gluconic acid production reduces rhizosphere pH, inhibiting flg22-binding to the plant receptor FLS2, reducing root immunity, and facilitating colonization [92]. These properties align with the previously reported plant-beneficial and rhizosphere-colonizing properties of *P. capeferrum* IsoF [3, 15–18].

Because adaptive traits in *Pseudomonas* are frequently encoded within GEIs, we hypothesized that the strong colonization and biocontrol capabilities of IsoF may be rooted in its accessory genome [12, 93]. Comparative analysis revealed that IsoF appears to carry a distinctive combination of GEIs, acting as a functionally complementary repertoire, with these GEIs being present as isolated traits in other plant- and rhizosphere-associated PGPR strains, including *P. capeferrum* WCS358, *P. alloputida* F1, *P. putida* (sp.) GB-1, *P. alloputida* KT2440, *P. alloputida* B6-2, *Pseudomonas* sp. H2, and *P. putida* (sp.) W619. WCS358 induces ISR in *Arabidopsis thaliana* and suppresses plant diseases through siderophore-mediated competition [94]. F1 and GB-1 genomes contain genes implicated in bioremediation and plant growth stimulation [93, 95], while KT2440 promotes drought tolerance in tomato, plant growth, and protection against pathogens via a T6SS [5, 96, 97]. B6-2 and H2 possess bioremediation capabilities [98, 99], and W619 contributes to plant growth and bioremediation [100–102]. The presence of putative putrescine transporters within GEIs of IsoF supports its close association with the rhizosphere, as putrescine, a common component of root exudates, promotes *Pseudomonas* root colonization [2, 103]. The presence of predicted bioremediation-related elements, including plastic degradation loci homologous to those in the polyurethane-degrading strain TDA1 [104], further expands the ecological breadth of this accessory genome.

Few virulence factors were identified within the GEIs of IsoF, supporting the view that these elements primarily enhance competitiveness and niche adaptation rather than pathogenicity (Table S10). This aligns with an emerging perspective that GEIs in environmental *Pseudomonas* function as ecological innovation modules. For instance, siderophore-related genes within two IsoF GEIs may promote plant protection by depleting available iron and inhibiting the growth of phytopathogens, analogous to WCS358 [2, 87–89]. A homologue of the exolysin-encoding gene *exlAB,* previously reported to contribute to insect lethality, may support IsoF’s biocontrol activity [105]. IsoF also encodes several secretion systems (Table S11), including T5SSs (T5aSS and T5bSS) and a complete T6SS cluster, which likely provide advantages against microbial competitors in the rhizosphere [5, 106]. The previously described IsoF T4BSS (*kib* cluster, GEI 15) functions as an efficient interbacterial killing system against phytopathogens such as *R. solenacearum* [3]. Notably, our rhizosphere assays demonstrate that deletion of the *kib* island reduces persistence under tomato-associated conditions, providing experimental evidence that a horizontally acquired GEI can directly enhances fitness under these conditions.

*Pseudomonas* species produce diverse CLPs, which support plant health by triggering ISR and antagonism against soil-borne pathogens [80, 81, 86]. In IsoF, the *ppu* QS system (GEI 6) regulates the putisolvin biosynthetic machinery (GEI 32), which contributes to swarming motility, antifungal activity, and biofilm dispersal [3, 17, 51, 80]. Although acquired independently, these loci appear functionally integrated, illustrating how distinct HGT events can be incorporated into regulatory networks that optimize ecological performance.

Altogether, the traits encoded by IsoF GEIs, particularly the *kib*, *pso,* and *ppu* clusters, may support rhizosphere colonization and ecological fitness. Strikingly, this specific island combination is restricted to only six soil-associated *P. capeferrum* strains worldwide, including *P. capeferrum* strains SWRI59 and SWRI68 (Iran) [81], *Pseudomonas* sp. Irchel s3f19 (Switzerland) [82], *P. capeferrum* B21–047 (Canada) [83], *P. capeferrum* HN2–3 (China) [80], and IsoF (Fig. 3, Fig. 5, Fig. S5). Two of these strains exhibit reported biocontrol activity: B21-047 moderately inhibits lettuce bacterial pathogens (*Pectobacterium carotovoum*, *P. cichorii,* and *X. hortorum* pv. *vitians*) [83] and *P. capeferrum* HN2-3 suppresses *Phytophtora capsica* zoospores via a highly homologous *pso* cluster to that of IsoF [80] (Figs 3 and 5, Fig. S5). This raises the possibility that IsoF may also exert antagonistic activity against these bacterial and oomycete phytopathogens due to the genomic similarities among these strains. Whether the antibacterial properties of B21-047 rely on its *kib* locus homolog remains to be determined. Nevertheless, the shared island architecture is consistent with a potentially conserved ecological strategy within this lineage.

Interestingly, only the *kib* cluster affected rhizosphere persistence of IsoF, while *ppu* and *pso* deletions had no detectable effect, despite the impaired swarming motility exhibited by these mutants on plate. These results indicate that the *ppu* QS system does not regulate the IsoF T4BSS (Fig. 6), consistent with the absence of QS-mediated control of the T4BSS in *L. pneumophila* and in contrast to the regulation reported for T4ASSs [107]. Collectively, our findings support a model in which GEIs act as modular ecological determinants that enable rapid niche specialization. In IsoF, the coordinated presence of multiple horizontally acquired islands has shaped a lineage-specific repertoire of traits associated with life in the rhizosphere. Among these, T4BSS makes a measurable contribution to rhizosphere persistence, whereas the pso and *ppu* systems provide additional phenotypes that may be relevant to ecological fitness under specific environmental conditions. More broadly, these results highlight GEIs as active drivers of microbial niche ecology rather than passive components of the accessory genome.

## Supplementary Material

Supplementary_material_ycag231

## Data Availability

The data underlying this article are available in the article and in its online supplementary material. The IsoF genome is found under the NCBI accession number NZ_CP072013.

## References

[1] Arora NK . Plant microbe Symbiosis: fundamentals and advances. *Plant Microbe Symbiosis Fundam Adv* 2013;1:1–459. 10.1007/978-81-322-1287-4

[2] Lugtenberg B, Kamilova F. Plant-growth-promoting rhizobacteria. *Ann Rev Microbiol* 2009;63:541–56. 10.1146/annurev.micro.62.081307.162918 19575558

[3] Purtschert-Montenegro G, Cárcamo-Oyarce G, Pinto-Carbó M, et al. *Pseudomonas putida* mediates bacterial killing, biofilm invasion and biocontrol with a type IVB secretion system. *Nat Microbiol* 2022;7:1547–57. 10.1038/s41564-022-01209-6 PMC9519443 36123439

[4] Backer R, Rokem JS, Ilangumaran G, et al. Plant growth-promoting rhizobacteria: context, mechanisms of action, and roadmap to commercialization of biostimulants for sustainable agriculture. *Front Plant Sci* 2018;9:1–17. 10.3389/fpls.2018.01473 PMC6206271 30405652

[5] Bernal P, Allsopp LP, Filloux A, et al. The *Pseudomonas putida* T6SS is a plant warden against phytopathogens. *ISME J* 2017;11:972–87. 10.1038/ismej.2016.169 PMC5363822 28045455

[6] Chiniquy D, Barnes EM, Zhou J, et al. Microbial community field surveys reveal abundant *Pseudomonas* population in sorghum rhizosphere composed of many closely related phylotypes. *Front Microbiol* 2021;12:12. 10.3389/fmicb.2021.598180 PMC7985074 33767674

[7] Zboralski A, Filion M. Genetic factors involved in rhizosphere colonization by phytobeneficial *Pseudomonas* spp. *Comput Struct Biotechnol J* 2020;18:3539–54. 10.1016/j.csbj.2020.11.025 PMC7711191 33304453

[8] Loper JE, Hassan KA, Mavrodi DV, et al. Comparative genomics of plant-associated *Pseudomonas* spp.: insights into diversity and inheritance of traits involved in multitrophic interactions. *PLoS Genet* 2012;8:e1002784. 10.1371/journal.pgen.1002784 PMC3390384 22792073

[9] Soucy SM, Huang J, Gogarten JP. Horizontal gene transfer: building the web of life. *Nat Publ Gr* 2015;16:472–82. 10.1038/nrg3962 26184597

[10] Dobrindt U, Hochhut B, Hentschel U, et al. Genomic islands in pathogenic and environmental microorganisms. *Nat Rev Microbiol* 2004;2:414–24. 10.1038/nrmicro884 15100694

[11] Ozer EA, Allen JP, Hauser AR. Characterization of the core and accessory genomes of *Pseudomonas aeruginosa* using bioinformatic tools spine and AGEnt. *BMC Genomics* 2014;15:737. 10.1186/1471-2164-15-737 PMC4155085 25168460

[12] Melnyk RA, Hossain SS, Haney CH. Convergent gain and loss of genomic islands drive lifestyle changes in plant-associated *Pseudomonas*. *ISME J* 2019;13:1575–88. 10.1038/s41396-019-0372-5 PMC6776051 30787396

[13] Kasai-Maita H, Hirakawa H, Nakamura Y, et al. Commonalities and differences among symbiosis islands of three *Mesorhizobium loti* strains. *Microbes Environ* 2013;28:275–8. 10.1264/jsme2.ME12201 PMC4070662 23666538

[14] Watanabe Y, Ishiga Y, Sakata N. The role of genomic islands in the pathogenicity and evolution of plant-pathogenic Gammaproteobacteria. *Microorganisms* 2025;13:13. 10.3390/microorganisms13081803 PMC12388829 40871307

[15] Steidle A, Allesen-Holm M, Riedel K, et al. Identification and characterization of an N-acylhomoserine lactone-dependent quorum-sensing system in *Pseudomonas putida* strain IsoF. *Appl Environ Microbiol* 2002;68:6371–82. 10.1128/AEM.68.12.6371-6382.2002 PMC134430 12450862

[16] Arevalo-Ferro C, Reil G, Görg A, et al. Biofilm formation of *Pseudomonas putida* IsoF: the role of quorum sensing as assessed by proteomics. *Syst Appl Microbiol* 2005;28:87–114. 10.1016/j.syapm.2004.10.005 15830802

[17] Cárcamo-Oyarce G, Lumjiaktase P, Kümmerli R, et al. Quorum sensing triggers the stochastic escape of individual cells from *Pseudomonas putida* biofilms. *Nat Commun* 2015;6:5945. 10.1038/ncomms6945 PMC4309448 25592773

[18] Schuhegger R, Ihring A, Gantner S, et al. Induction of systemic resistance in tomato by N-acyl-L-homoserine lactone-producing rhizosphere bacteria. *Plant Cell Environ* 2006;29:909–18. 10.1111/j.1365-3040.2005.01471.x 17087474

[19] Alanjary M, Steinke K, Ziemert N. AutoMLST: an automated web server for generating multi-locus species trees highlighting natural product potential. *Nucleic Acids Res* 2019;47:W276–82. 10.1093/NAR/GKZ282 PMC6602446 30997504

[20] Edler D, Klein J, Antonelli A, et al. raxmlGUI 2.0: a graphical interface and toolkit for phylogenetic analyses using RAxML. *Methods Ecol Evol* 2021;12:373–7. 10.1111/2041-210X.13512

[21] Olson RD, Assaf R, Brettin T, et al. Introducing the bacterial and viral bioinformatics resource center (BV-BRC): a resource combining PATRIC, IRD and ViPR. *Nucleic Acids Res* 2023;51:D678–89. 10.1093/nar/gkac1003 PMC9825582 36350631

[22] van den Belt M, Gilchrist C, Booth TJ, et al. CAGECAT: the CompArative GEne cluster analysis toolbox for rapid search and visualisation of homologous gene clusters. *BMC Bioinformatics* 2023;24:181. 10.1186/s12859-023-05311-2 PMC10155394 37131131

[23] Blin K, Shaw S, Kloosterman AM, et al. AntiSMASH 6.0: improving cluster detection and comparison capabilities. *Nucleic Acids Res* 2021;49:W29–35. 10.1093/nar/gkab335 PMC8262755 33978755

[24] Girard L, Geudens N, Pauwels B, et al. Transporter gene-mediated typing for detection and genome mining of lipopeptide-producing *pseudomonas*. *Appl Environ Microbiol* 2022;88:88. 10.1128/AEM.01869-21 PMC8788793 34731056

[25] Edgar RC . MUSCLE: multiple sequence alignment with high accuracy and high throughput. *Nucleic Acids Res* 2004;32:1792–7. 10.1093/nar/gkh340 PMC390337 15034147

[26] Waterhouse AM, Procter JB, Martin DMA, et al. Jalview version 2-a multiple sequence alignment editor and analysis workbench. *Bioinformatics* 2009;25:1189–91. 10.1093/bioinformatics/btp033 PMC2672624 19151095

[27] Needham DM, Poirier C, Bachy C, et al. The microbiome of a bacterivorous marine choanoflagellate contains a resource-demanding obligate bacterial associate. *Nat Microbiol* 2022;7:1466–79. 10.1038/s41564-022-01174-0 PMC9418006 35970961

[28] Price MN, Dehal PS, Arkin AP. FastTree 2 - approximately maximum-likelihood trees for large alignments. *PLoS One* 2010;5:5. 10.1371/journal.pone.0009490 PMC2835736 20224823

[29] Katoh K, Standley DM. MAFFT multiple sequence alignment software version 7: improvements in performance and usability. *Mol Biol Evol* 2013;30:772–80. 10.1093/molbev/mst010 PMC3603318 23329690

[30] Letunic I, Bork P. Interactive tree of life (iTOL) v6: recent updates to the phylogenetic tree display and annotation tool. *Nucleic Acids Res* 2024;52:W78–82. 10.1093/nar/gkae268 PMC11223838 38613393

[31] Grant JR, Enns E, Marinier E, et al. Proksee: In-depth characterization and visualization of bacterial genomes. *Nucleic Acids Res* 2023;51:W484–92. 10.1093/nar/gkad326 PMC10320063 37140037

[32] Soares SC, Geyik H, Ramos RTJ, et al. GIPSy: genomic island prediction software. *J Biotechnol* 2016;232:2–11. 10.1016/j.jbiotec.2015.09.008 26376473

[33] Bertelli C, Laird MR, Williams KP, et al. IslandViewer 4: expanded prediction of genomic islands for larger-scale datasets. *Nucleic Acids Res* 2017;45:W30–5. 10.1093/nar/gkx343 PMC5570257 28472413

[34] Liu M, Li X, Xie Y, et al. ICEberg 2.0: an updated database of bacterial integrative and conjugative elements. *Nucleic Acids Res* 2019;47:D660–5. 10.1093/nar/gky1123 PMC6323972 30407568

[35] Arndt D, Grant JR, Marcu A, et al. PHASTER: a better, faster version of the PHAST phage search tool. *Nucleic Acids Res* 2016;44:W16–21. 10.1093/NAR/GKW387 PMC4987931 27141966

[36] Dong MJ, Luo H, Gao F. Ori-finder 2022: a comprehensive web server for prediction and analysis of bacterial replication origins. *Genomics Proteomics Bioinformatics* 2022;20:1207–13. 10.1016/j.gpb.2022.10.002 PMC10225481 36257484

[37] Abby SS, Cury J, Guglielmini J, et al. Identification of protein secretion systems in bacterial genomes. *Sci Rep* 2016;6:1–14. 10.1038/srep23080 PMC4793230 26979785

[38] Néron B, Denise R, Coluzzi C, et al. MacSyFinder v2: improved modelling and search engine to identify molecular systems in genomes. *Peer Community J* 2023;3:3. 10.24072/pcjournal.250

[39] Liu B, Zheng D, Jin Q, et al. VFDB 2019: a comparative pathogenomic platform with an interactive web interface. *Nucleic Acids Res* 2019;47:D687–92. 10.1093/NAR/GKY1080 PMC6324032 30395255

[40] Cumsille A, Durán RE, Rodríguez-Delherbe A, et al. GenoVi, an open-source automated circular genome visualizer for bacteria and archaea. *PLoS Comput Biol* 2023;19:e1010998. 10.1371/JOURNAL.PCBI.1010998 PMC10104344 37014908

[41] Zhang Y, Guan J, Li C, et al. DeepSecE: a deep-learning-based framework for multiclass prediction of secreted proteins in gram-negative bacteria. *Research* 2023;6:6. 10.34133/research.0258 PMC10599158 37886621

[42] Clark DJ, Maaløe O. DNA replication and the division cycle in *Escherichia coli*. *J Mol Biol* 1967;23:99–112. 10.1016/S0022-2836(67)80070-6

[43] Flannagan RS, Linn T, Valvano MA. A system for the construction of targeted unmarked gene deletions in the genus *Burkholderia*. *Environ Microbiol* 2008;10:1652–60. 10.1111/j.1462-2920.2008.01576.x 18341581

[44] Choi KH, Schweizer HP. Mini-Tn7 insertion in bacteria with single attTn7 sites: example *Pseudomonas aeruginosa*. *Nat Protoc* 2006;1:153–61. 10.1038/nprot.2006.24 17406227

[45] Aguilar C, Schmid N, Lardi M, et al. The IclR-family regulator BapR controls biofilm formation in *B. Cenocepacia* H111. *PLoS One* 2014;9:1–7. 10.1371/journal.pone.0092920 PMC3962473 24658785

[46] Huber B, Riedel K, Köthe M, et al. Genetic analysis of functions involved in the late stages of biofilm development in *Burkholderia cepacia* H111. *Mol Microbiol* 2002;46:411–26. 10.1046/j.1365-2958.2002.03182.x 12406218

[47] Schindelin J, Arganda-Carreras I, Frise E, et al. Fiji: an open-source platform for biological-image analysis. *Nat Methods* 2012;9:676–82. 10.1038/nmeth.2019 PMC3855844 22743772

[48] Eberl L, Winson MK, Sternberg C, et al. Involvement of N-acyl-l-homoserine lactone autoinducers in controlling the multicellular behaviour of *Serratia liquefaciens*. *Mol Microbiol* 1996;20:127–36. 10.1111/j.1365-2958.1996.tb02495.x 8861211

[49] Mannweiler O, Pinto-Carbo M, Lardi M, et al. Investigation of *Burkholderia cepacia* complex methylomes via single-molecule, real-time sequencing and mutant analysis. *J Bacteriol* 2021;203:e0068320. 10.1128/JB.00683-20 PMC8316024 33753468

[50] Agnoli K, Schwager S, Uehlinger S, et al. Exposing the third chromosome of *Burkholderia cepacia* complex strains as a virulence plasmid. *Mol Microbiol* 2012;83:362–78. 10.1111/j.1365-2958.2011.07937.x 22171913

[51] Steidle A, Sigl K, Schuhegger R, et al. Visualization of N-acylhomoserine lactone-mediated cell-cell communication between bacteria colonizing the tomato rhizosphere. *Appl Environ Microbiol* 2001;67:5761–70. 10.1128/AEM.67.12.5761-5770.2001 PMC93370 11722933

[52] Molina-Santiago C, Udaondo Z, Gómez-Lozano M, et al. Global transcriptional response of solvent-sensitive and solvent-tolerant *Pseudomonas putida* strains exposed to toluene. *Environ Microbiol* 2017;19:645–58. 10.1111/1462-2920.13585 27768818

[53] Udaondo Z, Molina L, Segura A, et al. Analysis of the core genome and pangenome of *pseudomonas putida*. *Environ Microbiol* 2016;18:3268–83. 10.1111/1462-2920.13015 26261031

[54] Boyd EF, Almagro-Moreno S, Parent MA. Genomic islands are dynamic, ancient integrative elements in bacterial evolution. *Trends Microbiol* 2009;17:47–53. 10.1016/J.TIM.2008.11.003 19162481

[55] Aguilar-Barajas E, Díaz-Pérez C, Ramírez-Díaz MI, et al. Bacterial transport of sulfate, molybdate, and related oxyanions. *BioMetals* 2011;24:687–707. 10.1007/S10534-011-9421-X 21301930

[56] Igarashi K, Kashiwagi K. Polyamine modulon in *Escherichia coli*: genes involved in the stimulation of cell growth by polyamines. *J Biochem* 2006;139:11–6. 10.1093/jb/mvj020 16428314

[57] Rodríguez MP, Melo C, Jiménez E, et al. Glyphosate bioremediation through the sarcosine oxidase pathway mediated by *Lysinibacillus sphaericus* in soils cultivated with potatoes. *Agriculture* 2019;9:9. 10.3390/agriculture9100217

[58] Danhorn T, Fuqua C. Biofilm formation by plant-associated bacteria. *Ann Rev Microbiol* 2007;61:401–22. 10.1146/annurev.micro.61.080706.093316 17506679

[59] Hacker J, Kaper JB. Pathogenicity islands and the evolution of microbes. *Ann Rev Microbiol* 2000;54:641–79. 10.1146/annurev.micro.54.1.641 11018140

[60] Haas D, Défago G. Biological control of soil-borne pathogens by fluorescent pseudomonads. *Nat Rev Microbiol* 2005;3:307–19. 10.1038/nrmicro1129 15759041

[61] Brott AS, Clarke AJ. Peptidoglycan O-acetylation as a virulence factor: its effect on lysozyme in the innate immune system. *Antibiotics* 2019;8:94. 10.3390/antibiotics8030094 PMC6783866 31323733

[62] Moynihan PJ, Clarke AJ. O-acetylated peptidoglycan: controlling the activity of bacterial autolysins and lytic enzymes of innate immune systems. *Int J Biochem Cell Biol* 2011;43:1655–9. 10.1016/J.BIOCEL.2011.08.007 21889603

[63] Vasu K, Nagaraja V. Diverse functions of restriction-modification systems in addition to cellular defense. *Microbiol Mol Biol Rev* 2013;77:53–72. 10.1128/mmbr.00044-12 PMC3591985 23471617

[64] Cotter PD, Ross RP, Hill C. Bacteriocins-a viable alternative to antibiotics? *Nat Rev Microbiol* 2013;11:95–105. 10.1038/nrmicro2937 23268227

[65] Klein TA, Ahmad S, Whitney JC. Contact-dependent interbacterial antagonism mediated by protein secretion machines. *Trends Microbiol* 2020;28:387–400. 10.1016/j.tim.2020.01.003 32298616

[66] Chen L, Zou Y, She P, et al. Composition, function, and regulation of T6SS in *Pseudomonas aeruginosa*. *Microbiol Res* 2015;172:19–25. 10.1016/j.micres.2015.01.004 25721475

[67] Xin XF, Kvitko B, He SY. *Pseudomonas syringae*: what it takes to be a pathogen. *Nat Rev Microbiol* 2018;16:316–28. 10.1038/nrmicro.2018.17 PMC5972017 29479077

[68] Blondel CJ, Jiménez JC, Contreras I, et al. Comparative genomic analysis uncovers 3 novel loci encoding type six secretion systems differentially distributed in *salmonella* serotypes. *BMC Genomics* 2009;10:1–17. 10.1186/1471-2164-10-354 PMC2907695 19653904

[69] Journet L, Cascales E. The type VI secretion system in *Escherichia coli* and related species. *EcoSal Plus* 2016;7:7. 10.1128/ecosalplus.esp-0009-2015 PMC11575709 27223818

[70] Labbate M, Orata FD, Petty NK, et al. A genomic island in *vibrio cholerae* with VPI-1 site-specific recombination characteristics contains CRISPR-Cas and type VI secretion modules. *Sci Rep* 2016;6:1–7. 10.1038/srep36891 PMC5109276 27845364

[71] Guglielmini J, De La Cruz F, Rocha EPC. Evolution of conjugation and type IV secretion systems. *Mol Biol Evol* 2013;30:315–31. 10.1093/molbev/mss221 PMC3548315 22977114

[72] Guglielmini J, Néron B, Abby SS, et al. Key components of the eight classes of type IV secretion systems involved in bacterial conjugation or protein secretion. *Nucleic Acids Res* 2014;42:5715–27. 10.1093/nar/gku194 PMC4027160 24623814

[73] Lemarignier M, Savin C, Torres IR, et al. A type IVB secretion system contributes to the pathogenicity of Yersinia pseudotuberculosis strains responsible for the Far East scarlet-like fever. bioRxiv. 2024. 10.1101/2024.06.14.598817

[74] Barretto LAF, Fowler CC. Identification of a putative T6SS immunity islet in *Salmonella typhi*. *Pathogens* 2020;9:1–15. 10.3390/pathogens9070559 PMC7400221 32664482

[75] Azhieh A, Hernandez P, Anderson AC, et al. Rapidly evolving orphan immunity genes protect human gut bacteria from intoxication by the type VI secretion system. bioRxiv. 2025. 10.1101/2025.05.03.651265

[76] Kim H, Kubori T, Yamazaki K, et al. Structural basis for effector protein recognition by the dot/Icm type IVB coupling protein complex. *Nat Commun* 2020;11:1–11. 10.1038/s41467-020-16397-0 PMC7251119 32457311

[77] Liu M, Wang HHH, Liu Y, et al. The phospholipase effector Tle1Vc promotes *Vibrio cholerae* virulence by killing competitors and impacting gene expression. *Gut Microbes* 2023;15:15. 10.1080/19490976.2023.2241204 PMC10395195 37526354

[78] Jung BK, Ibal JC, Pham HQ, et al. Quorum sensing system affects the plant growth promotion traits of *Serratia fonticola* GS2. *Front Microbiol* 2020;11:11. 10.3389/fmicb.2020.536865 PMC7720635 33329415

[79] Zhuang X, Liu Y, Fang N, et al. Quorum sensing improves the plant growth-promoting ability of *Stenotrophomonas rhizophila* under saline-alkaline stress by enhancing its environmental adaptability. *Front Microbiol* 2023;14:14. 10.3389/fmicb.2023.1155081 PMC10126360 37113227

[80] Sheng J, Qin X, Yang X, et al. The biocontrol roles of cyclic lipopeptide putisolvin produced from *pseudomonas capeferrum* HN2-3 on the *phytophthora* blight disease in cucumbers. *J Plant Dis Prot* 2024;131:423–32. 10.1007/s41348-024-00874-5

[81] Girard L, Lood C, Höfte M, et al. The ever-expanding *Pseudomonas* genus: description of 43 new species and partition of the pseudomonas putida group. *Microorganisms* 2021;9:1–24. 10.3390/microorganisms9081766 PMC8401041 34442845

[82] Butaite E, Baumgartner M, Wyder S, et al. Siderophore cheating and cheating resistance shape competition for iron in soil and freshwater *pseudomonas* communities. *Nat Commun* 2017;8:414. 10.1038/s41467-017-00509-4 PMC5583256 28871205

[83] Zboralski A, Biessy A, Ciotola M, et al. Harnessing the genomic diversity of *Pseudomonas* strains against lettuce bacterial pathogens. *Front Microbiol* 2022;13:13. 10.3389/fmicb.2022.1038888 PMC9814014 36620043

[84] Armenova N, Tsigoriyna L, Arsov A, et al. Antifungal biocontrol in sustainable crop protection: microbial lipopeptides, polyketides, and plant-derived agents. *J Fungi* 2025;12:22. 10.3390/jof12010022 PMC12843204 41590434

[85] Kruijt M, Tran H, Raaijmakers JM. Functional, genetic and chemical characterization of biosurfactants produced by plant growth-promoting *pseudomonas putida* 267. *J Appl Microbiol* 2009;107:546–56. 10.1111/j.1365-2672.2009.04244.x 19302489

[86] Geudens N, Martins JC. Cyclic lipodepsipeptides from *pseudomonas* spp. - biological Swiss-Army knives. *Front Microbiol* 2018;9:1–18. 10.3389/fmicb.2018.01867 PMC6104475 30158910

[87] Berendsen RL, van Verk MC, Stringlis IA, et al. Unearthing the genomes of plant-beneficial *pseudomonas* model strains WCS358, WCS374 and WCS417. *BMC Genomics* 2015;16:539. 10.1186/s12864-015-1632-z PMC4509608 26198432

[88] Geels FP, Schippers B. Reduction of yield depressions in high frequency potato cropping soil after seed tuber treatments with antagonistic fluorescent *Pseudomonas* spp. *J Phytopathol* 1983;108:207–14. 10.1111/j.1439-0434.1983.tb00580.x

[89] Geels FP, Schippers B. Selection of antagonistic fluorescent *Pseudomonas* spp. and their root colonization and persistence following treatment of seed potatoes. *J Phytopathol* 1983;108:193–206. 10.1111/j.1439-0434.1983.tb00579.x

[90] Budzikiewicz H . Siderophores of the Pseudomonadaceae *sensu stricto* (fluorescent and non-fluorescent *pseudomonas* spp.). *ChemInform* 2005;36:36. 10.1002/chin.200531214 15079896

[91] Hansen ML, Wibowo M, Jarmusch SA, et al. Sequential interspecies interactions affect production of antimicrobial secondary metabolites in *pseudomonas protegens* DTU9.1. *ISME J* 2022;16:2680–90. 10.1038/s41396-022-01322-8 PMC9666462 36123523

[92] Yu K, Liu Y, Tichelaar R, et al. Rhizosphere-associated *Pseudomonas* suppress local root immune responses by gluconic acid-mediated lowering of environmental pH. *Curr Biol* 2019;29:3913–3920.e4. 10.1016/j.cub.2019.09.015 31668625

[93] Wu X, Monchy S, Taghavi S, et al. Comparative genomics and functional analysis of niche-specific adaptation in *Pseudomonas putida*. *FEMS Microbiol Rev* 2011;35:299–323. 10.1111/j.1574-6976.2010.00249.x PMC3056050 20796030

[94] Meziane H, Van Der Sluis I, Van Loon LC, et al. Determinants of *Pseudomonas putida* WCS358 involved in inducing systemic resistance in plants. *Mol Plant Pathol* 2005;6:177–85. 10.1111/j.1364-3703.2005.00276.x 20565648

[95] Taghavi S, Garafola C, Monchy S, et al. Genome survey and characterization of endophytic bacteria exhibiting a beneficial effect on growth and development of poplar trees. *Appl Environ Microbiol* 2009;75:748–57. 10.1128/AEM.02239-08 PMC2632133 19060168

[96] Saglam A, Demiralay M, Colak DN, et al. *Pseudomonas putida* KT2440 induces drought tolerance during fruit ripening in tomato. *Bioagro* 2022;34:139–50. 10.51372/bioagro342.4

[97] Costa-Gutierrez SB, Raimondo EE, Vincent PA, et al. Importance of biofilm formation for promoting plant growth under salt stress in *Pseudomonas putida* KT2440. *J Basic Microbiol* 2023;63:1219–32. 10.1002/jobm.202300215 37537345

[98] Fan S, Ren H, Fu X, et al. Genome streamlining of *Pseudomonas putida* B6-2 for bioremediation. *mSystems* 2024;9:9. 10.1128/msystems.00845-24 PMC11658094 39530686

[99] Su J, Bai Y, Huang T, et al. Performance and microbial community of simultaneous denitrification and biomineralization in bioreactors. *Chem Eng Technol* 2019;42:637–44. 10.1002/ceat.201800310

[100] Weyens N, Truyens S, Dupae J, et al. Potential of the TCE-degrading endophyte *Pseudomonas putida* W619-TCE to improve plant growth and reduce TCE phytotoxicity and evapotranspiration in poplar cuttings. *Environ Pollut* 2010;158:2915–9. 10.1016/j.envpol.2010.06.004 20598789

[101] Passarelli-Araujo H, Jacobs SH, Franco GR, et al. Phylogenetic analysis and population structure of *Pseudomonas alloputida*. *Genomics* 2021;113:3762–73. 10.1016/j.ygeno.2021.09.008 34530104

[102] Keshavarz-Tohid V, Vacheron J, Dubost A, et al. Genomic, phylogenetic and catabolic re-assessment of the *Pseudomonas putida* clade supports the delineation of *Pseudomonas alloputida* sp. nov., *Pseudomonas inefficax* sp. nov., *Pseudomonas persica* sp. nov., and *Pseudomonas shiraz*. *Syst Appl Microbiol* 2019;42:468–80. 10.1016/j.syapm.2019.04.004 31122691

[103] Kuiper I, Bloemberg GV, Noreen S, et al. Increased uptake of putrescine in the rhizosphere inhibits competitive root colonization by *Pseudomonas fluorescens* strain WCS365. *Mol Plant-Microbe Interact* 2001;14:1096–104. 10.1094/MPMI.2001.14.9.1096 11551074

[104] Puiggené Ò, Espinosa MJC, Schlosser D, et al. Extracellular degradation of a polyurethane oligomer involving outer membrane vesicles and further insights on the degradation of 2,4-diaminotoluene in *Pseudomonas capeferrum* TDA1. *Sci Rep* 2022;12:2666. 10.1038/s41598-022-06558-0 PMC8854710 35177693

[105] Job V, Gomez-Valero L, Renier A, et al. Genomic erosion and horizontal gene transfer shape functional differences of the ExlA toxin in *Pseudomonas* spp. *iScience* 2022;25:104596. 10.1016/j.isci.2022.104596 PMC9250014 35789842

[106] Fourie A, López JL, Sánchez-Gil JJ. et al. Bacterial family-specific enrichment and functions of secretion systems in the rhizosphere. bioRxiv. 2024; 2024–05.

[107] Allombert J, Jaboulay C, Michard C, et al. Deciphering *Legionella* effector delivery by Icm/dot secretion system reveals a new role for c-di-GMP signaling. *J Mol Biol* 2021;433:166985. 10.1016/j.jmb.2021.166985 33845084

[108] Davis JJ, Gerdes S, Olsen GJ, et al. PATtyFams: protein families for the microbial genomes in the PATRIC database. *Front Microbiol* 2016;7:7. 10.3389/fmicb.2016.00118 PMC4744870 26903996

