# Supplementary material for: Lineage-restricted genomic islands drive rhizosphere niche specialization in Pseudomonas capeferrum
Source: ISME Commun. 2026 Aug 23;6(1):ycag231. doi: 10.1093/ismeco/ycag231 (PMC13600407; doi:10.1093/ismeco/ycag231)
Supplement: Supplementary file 1 [file supplementary_material_ycag231.zip › Supplementary_Figures_Lopez-Carrasco_et_al_ycag231.pdf]

# Lineage-restricted genomic islands drive rhizosphere niche specialisation in *Pseudomonas capeferrum*

Miguel A. López-Carrasco[1], Gabriela Purtschert-Montenegro[1], Marta Pinto-Carbo[1], Gerardo Carcamo-Oyarce[1†], Ratchara Kalawong[1‡] and Leo Eberl1*

1 Department of Plant and Microbial biology, University of Zurich, Zurich, Switzerland

†Current address: Facultad de Ciencias Biológicas, Facultad de Medicina, Pontificia Universidad Católica de Chile.

‡Current address: Department of Microbiology, Faculty of Science, Mahidol University, Thailand.

*Correspondence to: leberl@botinst.uzh.ch

## Supplementary Figures

**Figure S1. IsoF genome. A.** Dot plot visualization of whole-genome synteny analysis of *Pseudomonas capeferrum* IsoF. The analysis was performed with Gepard and confirmed the integrity of the assembled chromosome. **B-C.** Two microsatellite regions were identified within the genome. One was located within the gene *aaeB* (coordinates: 1,673,785–1,673,798) (**B**), and the other within *psoB* (coordinates: 4,963,705–4,963,676) (**C**).

**Figure S2. Cluster of Orthologous Gene (COG) analysis.** COG classification of CDSs in the IsoF genome, including CDS within the corresponding GEIs. Analysis performed using Genovi. Total counts and percentage values for each category are indicated.

**Figure S3. Phylogenetic analysis of T4BSSs based on VirB4/DotO/TraU sequence homology. A.** The T4BSS of IsoF (red point) was localised in the type IV protein secretion systems of the mating-pair formation type I ($MPF_I$) subgroup (pT4SSi). **B.** A closer view of the pT4SSi branch situates the DotO component of the T4BSS of IsoF close to the T4BSS of the clinical isolates *P. putida* H8234 and *Yersinia pseudotuberculosis* IP31758.

**Figure S4. Analysis of genomic clusters associated with cyclic lipopeptides (CLPs) production.** Phylogenetic analysis of MacB protein sequences was performed to classify clusters to their corresponding CLP groups.

**Figure S5. Genomic comparison of GEIs of interest among selected *P. capeferrum* strains.** Genome alignment of the selected *P. capeferrum* strains and the GEI regions corresponding to the *ppu* QS system (**A,** GEI 6), the *pso* cluster (**B**, GEI 32) and the *kib* cluster (**C**, GEI 15) of IsoF. Analysis was performed using Proksee.

**Figure S6. Deletion mutants of IsoF GEIs.** Growth curve (**A**) and cell morphology (**B**) of mCherry-tagged deletion mutants of IsoF *ppu* (Δppu), *pso* (ΔpsoABC) and *kib* (ΔT4B) clusters were analysed. Bars correspond to 5 µm.

**Figure S7. IsoF is able to kill Gram-negative bacteria in a contact-dependent manner.** Dead cells (propidium iodide staining, red fluorescence) of selected strains in mixed competitions against IsoF::GFP (green) were counted under a confocal microscope, differentiating cells in contact or not with IsoF. No significant differences were observed in competitions against Gram-positive strains. Monocultures were used as controls. Bars correspond to 5 µm.

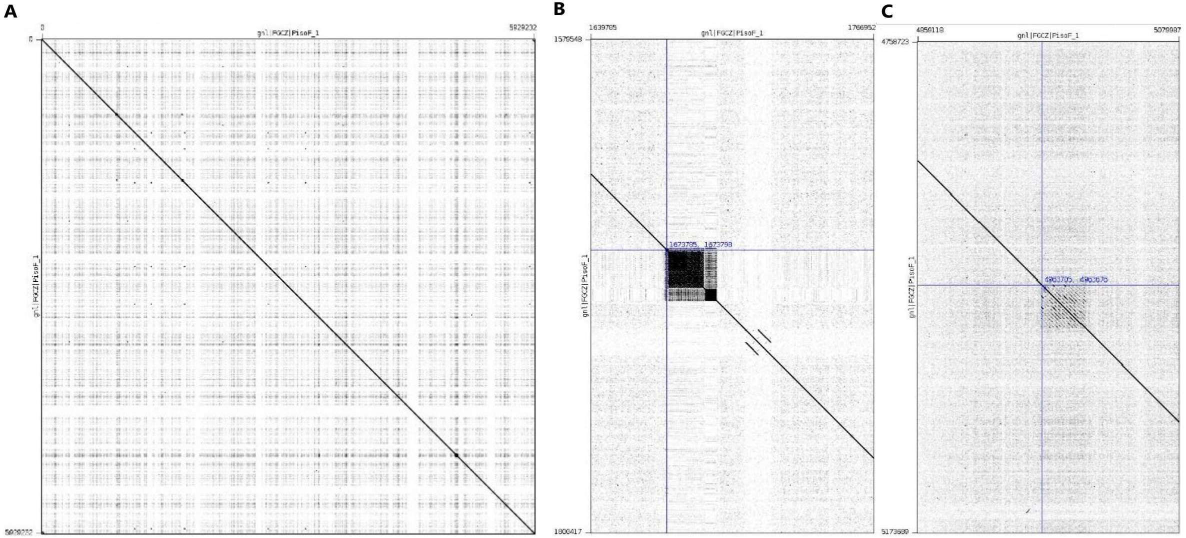


**Figure S1. IsoF genome. A.** Dot plot visualization of whole-genome synteny analysis of *Pseudomonas capeferrum* IsoF. The analysis was performed with Gepard and confirmed the integrity of the assembled chromosome. **B-C.** Two microsatellite regions were identified within the genome. One was located within the gene *aaeB* (coordinates: 1,673,785–1,673,798) (**B**), and the other within *psoB* (coordinates: 4,963,705–4,963,676) (**C**).

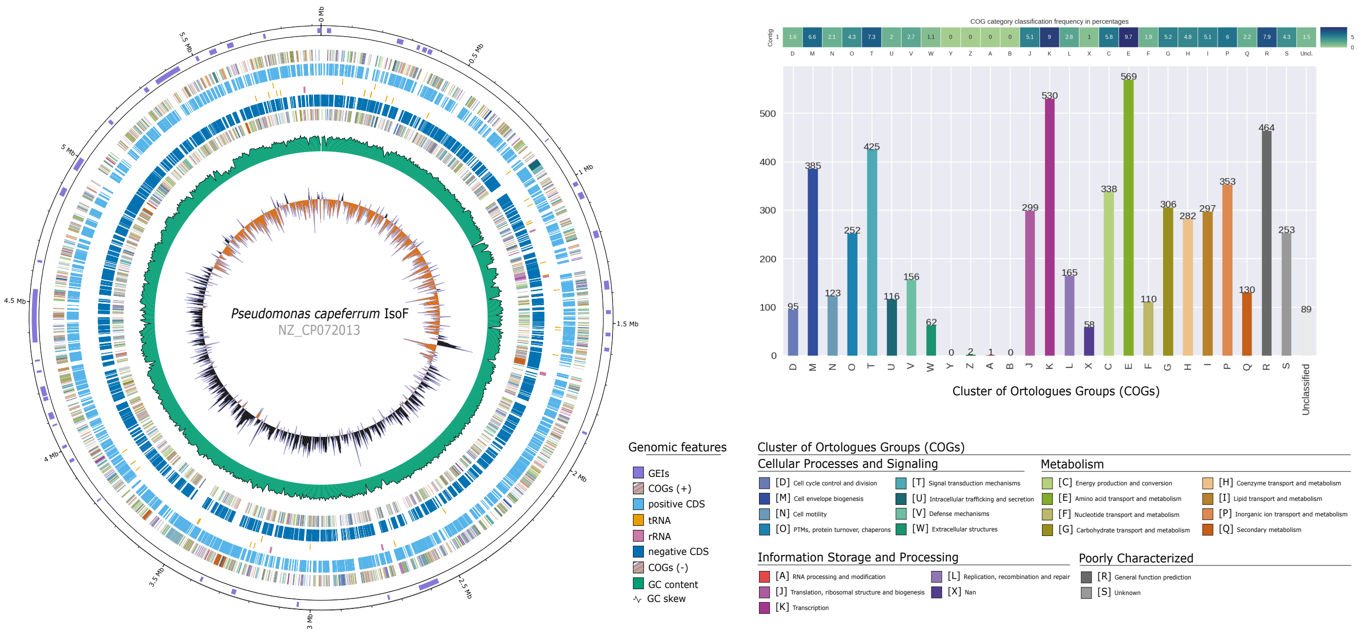


**Figure S2. Cluster of Orthologous Gene (COG) analysis.** COG classification of CDSs in the IsoF genome, including CDS within the corresponding GEIs. Analysis performed using Genovi. Total counts and percentage values for each category are indicated.

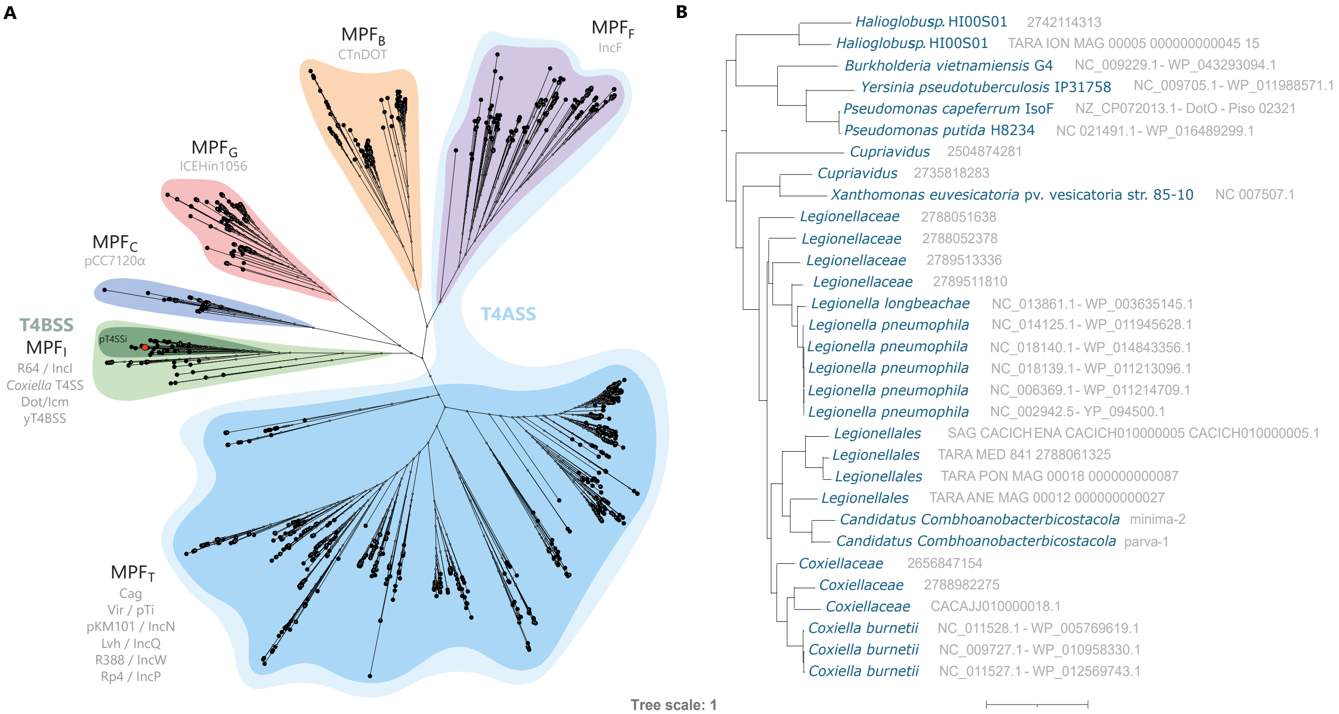


**Figure S3. Phylogenetic analysis of T4BSSs based on VirB4/DotO/TraU sequence homology. A.** The T4BSS of IsoF (red point) was localised in the type IV protein secretion systems of the mating-pair formation type I (MPFI) subgroup (pT4SSi). **B.** A closer view of the pT4SSi branch situates the DotO component of the T4BSS of IsoF close to the T4BSS of the clinical isolates *P. putida* H8234 and *Yersinia pseudotuberculosis* IP31758.

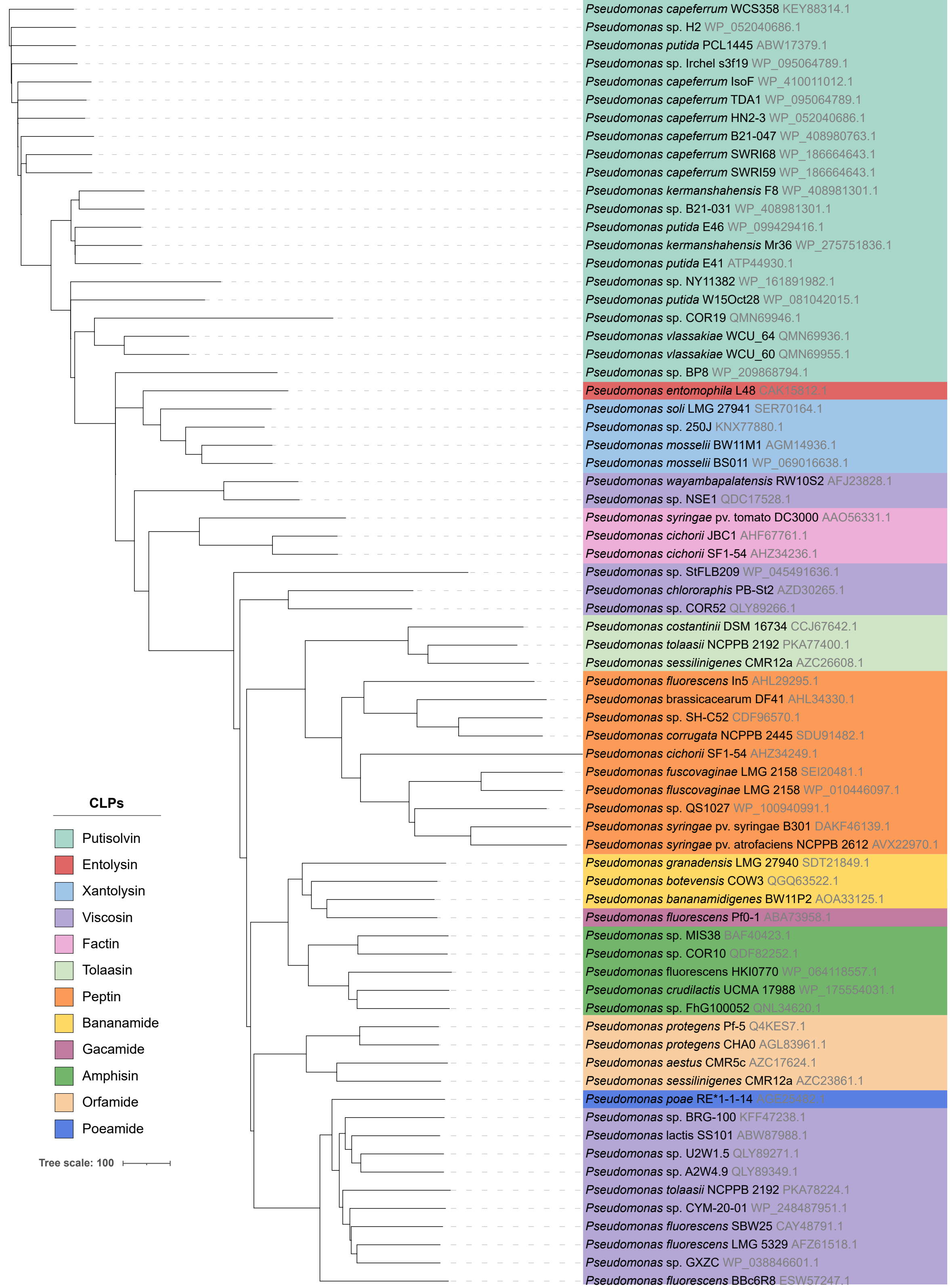


**Figure S4. Analysis of genomic clusters associated with cyclic lipopeptides (CLPs) production.** Phylogenetic analysis of MacB protein sequences was performed to classify clusters to their corresponding CLP groups.

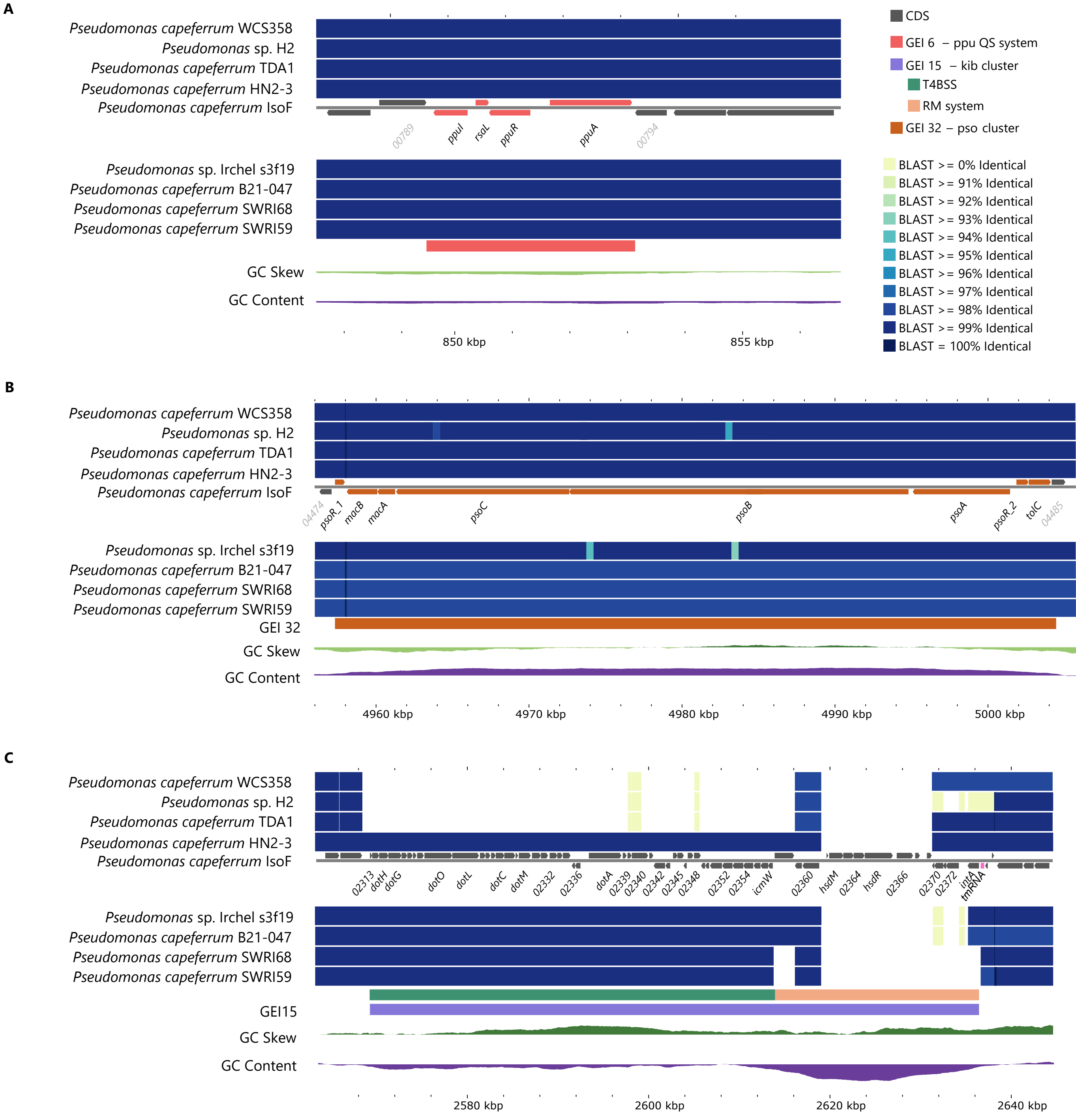


**Figure S5. Genomic comparison of GEIs of interest among selected *P. capeferrum* strains.** Genome alignment of the selected *P. capeferrum* strains and the GEI regions corresponding to the *ppu* QS system (**A,** GEI 6), the *pso* cluster (**B**, GEI 32) and the *kib* cluster (**C**, GEI 15) of IsoF. Analysis was performed using Proksee.

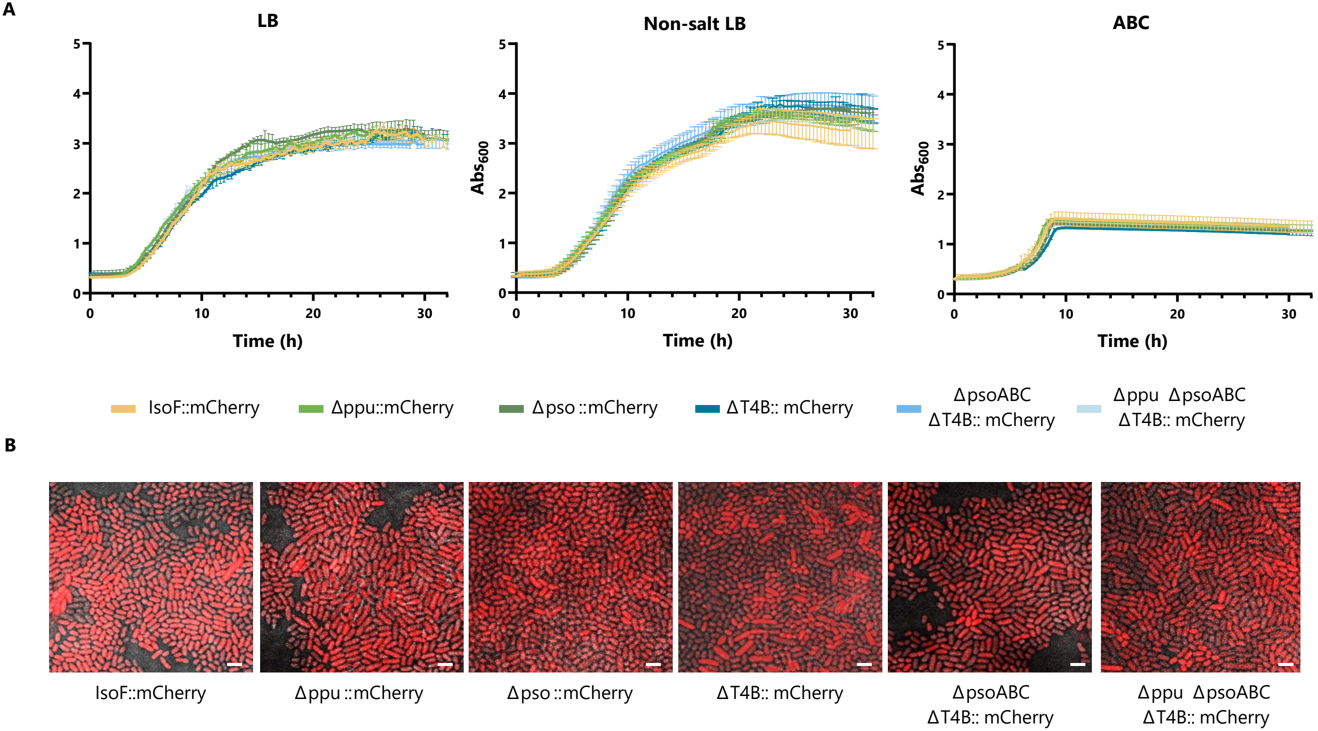


**Figure S6. Deletion mutants of IsoF GEIs.** Growth curve (**A**) and cell morphology (**B**) of mCherry-tagged deletion mutants of IsoF *ppu* (Δppu), *pso* (ΔpsoABC) and *kib* (ΔT4B) clusters were analysed. Bars correspond to 5 µm.

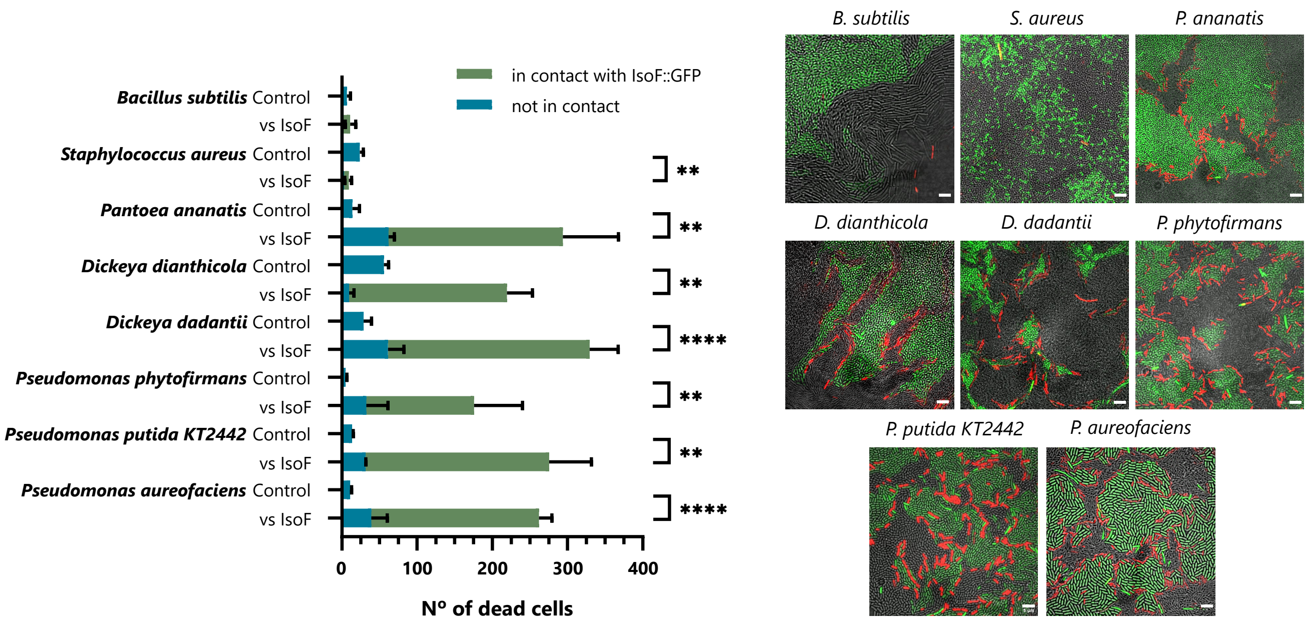


**Figure S7. IsoF is able to kill Gram-negative bacteria in a contact-dependent manner.** Dead cells (propidium iodide staining, red fluorescence) of selected strains in mixed competitions against IsoF::GFP (green) were counted under a confocal microscope, differentiating cells in contact or not with IsoF. No significant differences were observed in competitions against Gram-positive strains. Monocultures were used as controls. Bars correspond to 5 µm.
