# Supplementary material for: Lineage-restricted genomic islands drive rhizosphere niche specialization in Pseudomonas capeferrum
Source: ISME Commun. 2026 Aug 23;6(1):ycag231. doi: 10.1093/ismeco/ycag231 (PMC13600407; doi:10.1093/ismeco/ycag231)
Supplement: Supplementary file 1 [file supplementary_material_ycag231.zip › Supplementary_Text_Lopez-Carrasco_et_al_ycag231.docx]

**Lineage-restricted genomic islands drive rhizosphere niche specialisation in *Pseudomonas capeferrum***

Miguel A. López-Carrasco^1^, Gabriela Purtschert-Montenegro^1^, Marta Pinto-Carbo^1^,
Gerardo Carcamo-Oyarce^1†^, Ratchara Kalawong^1‡^ and Leo Eberl^1^*

^1^ Department of Plant and Microbial biology, University of Zurich, Zurich, Switzerland

^†^Current address: Facultad de Ciencias Biológicas, Facultad de Medicina, Pontificia Universidad Católica de Chile.

^‡^Current address: Department of Microbiology, Faculty of Science, Mahidol University, Thailand.

*Correspondence to: [leberl@botinst.uzh.ch](mailto:leberl@botinst.uzh.ch)

**Table of contents:**

1. Supplementary Methods
2. Supplementary Tables
3. Supplementary References

**1. Supplementary Methods**

**Genomic DNA extraction**

Genomic DNA (gDNA) of *P. capeferrum* IsoF used for sequencing was extracted using a previously described protocol [1] with the following modifications. After treatment with 5 M NaCl, 144 μl of protein precipitation solution from a Wizard® Genomic DNA Purification Kit (Promega, A1120) was added and samples were incubated on ice for 5 minutes. DNA pellets were washed and resuspended in 50 μl EB buffer (Qiagen). For Illumina sequencing, gDNA was extracted using the Gen Elute^TM^ Bacterial Genomic DNA Kit (Sigma-Aldrich). The purity and concentration of both gDNA preparations were evaluated using a Nanodrop spectrophotometer and the Qubit^TM^ dsDNA BR Assay Kit (ThermoFisher Scientific). Additionally, 1 μl of gDNA was run on a 1% agarose gel for visual assessment of integrity, prior to genome sequencing.

**Genome sequencing, assembly and annotation**

The genome of *P. capeferrum* IsoF was sequenced at the Functional Genomics Center Zurich (FGCZ). The DNA library was sequenced on a single SMRT cell using the PacBio RSII sequencing platform, and raw reads were assembled using the SMRT Portal assembly pipeline. To improve the accuracy of the assembly, quality-trimmed Illumina paired-end reads (150 bp) were mapped to the PacBio-derived contig using CLC Genomics Workbench. Subsequent polishing was performed with Pilon v1.24 [2], using the “fixed base settings” on programme "fix all". Automated genome annotation was performed with Prokka v1.14.6 [3], using *Pseudomonas alloputida* KT2440 as reference and followed by manual curation using Artemis [4]. Dot plot whole-genome synteny analysis was performed using Gepard [5].

**2. Supplementary Tables**

**Table S1.** Genome features of IsoF and eleven closely related *Pseudomonas* strains.

| **Strains** | **Size (bp)** | **GC%** | **CDS** | **16S rRNA** | **tRNA** | **Source** | **Origin** | **ID** | **Reference** |
| --- | --- | --- | --- | --- | --- | --- | --- | --- | --- |
| **IsoF** | 5,929,333 | 62.56 | 5242 | 7 | 77 | Rhizosphere | Switzerland | NZ_CP072013.1 | Purtschert-Montenegro et al. (2022)[7] |
| **Irchel s3f19** | 5,969,965 | 62.21 | 5370 | 1 | 63 | Soil | Switzerland | NZ_FYDW00000000.1 | Butaite et al. (2017)[8] |
| **H2** | 5,790,406 | 62.23 | 5177 | 1 | 59 | Wastewater | USA | NZ_JRPO00000000.1 | Loftie-Eaton et al. (2015)[9] |
| **WCS358** | 5,940,443 | 61.93 | 5309 | 4 | 70 | Potato root | Netherlands | NZ_JMIT00000000.1 | Geels & Schippers (1983)[10, 11] |
| **GB-1** | 6,078,430 | 61.94 | 5460 | 7 | 74 | Fresh water | USA | NC_010322.1 | Wu et al. (2011)[12] |
| **SWI36** | 6,172,256 | 61.83 | 5621 | 7 | 76 | Soil | USA | NZ_CP026675.1 | Unpublished |
| **JB** | 5,846,746 | 61.81 | 5309 | 6 | 72 | Contaminated soil | Czech Republic | NZ_CP016212.1 | Unpublished |
| **BIRD** **-1** | 5,731,541 | 61.74 | 5152 | 7 | 65 | Soil | Spain | NC_017530.1 | Matilla et al. (2010)[13] |
| **KT2440** | 6,181,873 | 61.52 | 5481 | 7 | 74 | Garden soil | Japan | NC_002947.4 | Nelson et al. (2002)[14] |
| **B6-2** | 6,377,271 | 61.56 | 5711 | 7 | 82 | Contaminated soil | China | NZ_CP015202.1 | Wang et al. (2021)[15] |
| **F1** | 5,959,964 | 61.86 | 5423 | 6 | 76 | Polluted creek | USA | NC_009512.1 | Unpublished |
| **W619** | 5,774,330 | 61.43 | 5292 | 7 | 75 | Cottonwood tree | USA | NC_010501.1 | Unpublished |

| **Table S2.** Strains and plasmids used in this study. | | | |
| --- | --- | --- | --- |
| **Strain** | **Characteristics** | | **Source / Reference** |
| ***E. coli*** | | | |
| **Dh5α** | F- Ф80d*lacZ*Δ*M15 recA1 endA1* Δ*(lacZYA-argF)*U169  *hsdR17(rK- mK+) thi-1 supE44 gyrA96 relA1* | | Hanahan (1983)[16] |
| **S17-1λpir** | *thi hsdR proA recA kan::Tn7* RP4-2-*tet*::Mu-1 integrant  (Tp^R^ Sm^R^) λpir | | Simon et al. (1983)[17] |
| ***P. capeferrum*** | | | |
| **IsoF *** | Wild type | | Steidle et al.  (2001)[18] |
| **ΔT4B *** | IsoF deletion mutant of part of the *kib* cluster, from *PisoF_02313* to *PisoF_02360* | | Purtschert-Montenegro  et al. (2022)[7] |
| **Δppu *** | IsoF deletion mutant of the *ppu* cluster, from *PisoF_00790 to PisoF_00794* | | This study |
| **ΔpsoABC *** | IsoF deletion mutant of elements of the *pso* cluster, from *PisoF_04479 to PisoF_04481* | | This study |
| **ΔT4B ΔpsoABC *** | IsoF deletion mutant of *pso* and part of the *kib* (*PisoF_02313-PisoF_02360*) clusters | | This study |
| **ΔT4B Δppu ΔpsoABC *** | IsoF deletion mutant of *pso*, *ppu* and part of the *kib* (*PisoF_02313-PisoF_02360*) clusters | | This study |
| **PL11** | IsoF deletion mutant of *psoA* | | Cárcamo-Oyarce  et al., (2015) [19] |
| **PL2** | Conditional *psoA* mutant; P*_rhaB_*-*psoA* | | Cárcamo-Oyarce  et al., (2015)[19] |
| *** Also labelled with mCherry using the pUC18T-mini-Tn*7*T-Gm-*mCherry* plasmid.** | | | This study |
| **Strains used in competitions *P. capeferrum*** | | | |
| ***Pseudomonas chlororaphis* subsp. aureofaciens ATCC13985 *** | | Wild type | Conway et al.,  (1956)[20] |
| ***Bacillus subtilis* subsp. subtilis str. 168** | | Wild-type | Laboratory collection |
| ***Staphylococcus aureus* DSM 20231** | | Wild type | Laboratory collection |
| ***Pantoea ananatis* DMS 30070** | | Wild type | DSMZ collection |
| ***Dickeya dadantii* DSM 18020** | | Wild type | DSMZ collection |
| ***Dickeya dianthicola* DSM 18054** | | Wild type | DSMZ collection |
| ***Paraburkholderia phytofirmans* DSM 17436** | | Wild type | Laboratory collection |
| ***Pseudomonas putida* KT2442** | | Wild type | Bernal et al. (2017)[21] |
| *** Also labelled with mCherry using the pUC18T-mini-Tn*7*T-Gm-*mCherry* plasmid.** | | | Purtschert-Montenegro  et al. (2022)[7] |
| ***Plasmids*** | | | |
| **pUC18T-mini-Tn*7*T- Gm-*mCherry*** | carrying mini Tn7(Gm) PA1/04/03-mCherry for mCherry  tagging | | Choi &  Schweizer (2006)[22] |
| **pUX-BF13** | tnsA-E; helper plasmid providing the Tn7 transposition  functions | | Koch et al.  (2001)[23] |
| **pGPI-SceI::TetAR** | Suicide plasmid with oriR6K, I-SceI restriction site, mob+; Tp^R^ Tc^R^ | | Purtschert-Montenegro  et al. (2022)[7] |
| **pGPI::TetAR-T4B** | pGPI-SceI-Tet plasmid with fused regions flanking the  *kib* cluster (*PisoF_02313* to *PisoF_02360*) | | Purtschert-Montenegro  et al. (2022)[7] |
| **pGPI::TetAR-ppu** | pGPI-SceI-Tet plasmid with fused regions flanking the  *ppu* cluster (*PisoF_00790* to *PisoF_00794*) | | This study |
| **pGPI::TetAR-psoABC** | pGPI-SceI-Tet plasmid with fused regions flanking the  *psoABC* genes (*PisoF_04479* to *PisoF_04481*) | | This study |
| **pDAIGm-SceI** | pDA17 plasmid with the *I-SceI* nuclease gene; Gm^R^ | | Flannagan et  al. (2008)[24] |
| **pRK2013** | Helper plasmid; RK2 derivative, *tra+ mob+ ori* ColE1;  Km^R^ | | Figurski &  Helinski (1979)[25] |

| **Table S3.** Primers used in this study | | |
| --- | --- | --- |
| **Primer** | **Sequence** | **Description/ Restriction site** |
| **Primers used for construction of markerless deletion of the *ppu* cluster** | | |
| dLppuFw | GCGCggtaccCATCCCTCATTTCGCTGTCA | KpnI |
| dLppuRv | GCGCccatggCGTGCGGCTGTTATATCAAT | NcoI |
| dLppuAFw | GCGCccatggCAATGTGAGCTTTACTGCC | NcoI |
| dLppuARv | GCGCgaattcGAATAAATCAATGCTGGTGG | EcoRI |
| *ppu*_out_F | GCGAAGAAGGCAAGGACTA | Checking *ppu* |
| *ppu*_out_R | TTTTTTTGCTCCAAGGCAG | Checking *ppu* |
| psoABC_up_F_XbaI | GCGCtctagaTCGGCAGGTCCTTCTGAT | XbaI |
| psoABC_up_R_KpnI | GCGCggtaccCAGCTTCATATCATTGTCTTCC | KpnI |
| psoABC_dn_F_HindIII | GCGCaagcttCATTGATGCGGGTGTAACCCAG | HindIII |
| psoABC_dn_R_XbaI | GCGCtctagaTATGCGAATCCCGAGAGAAAGTG | XbaI |
| psoABC_check_F | TCGCCCAGGATAGTGAAGTA | Checking *psoABC* |
| psoABC_check_R | TTTGAATTCGATCAGCAGCG | Checking *psoABC* |
| **Primers used to confirm markerless deletion of the *kib* cluster** | | |
| *kib*_out_F | ACGGCCAAGTTGAGTTACAA | Checking *kib* |
| *kib*_out_R | AATTTTACAGCCCAAAACCC | Checking *kib* |
| **Primers used to confirm markerless deletion of the *pso* cluster** | | |
| *pso*_out_F | ACGGCCAAGTTGAGTTACAA | Checking *pso* |
| *pso* _out_R | AATTTTACAGCCCAAAACCC | Checking *pso* |
| **Check primers of MCS of pGPI-SceI** | | |
| p44_pGPI_F | AGCTGATCCGGTGGATGAC | Checking pGPI |
| p45_pGPI_R | ACGGTTGTGGACAACAAGC | Checking pGPI |

| **Table S4.** Seedling counts per treatment and biological replicate | | | | | | |
| --- | --- | --- | --- | --- | --- | --- |
| **Biological replicate** | **IsoF::mCherry** | **Δppu::mCherry** | **Δpso::mCherry** | **ΔT4B::mCherry** | **ΔT4B Δpso::mCherry** | **ΔT4B Δppu Δpso::mCherry** |
| **1** | 6 | 6 | 6 | 6 | 6 | 6 |
| **2** | 6 | 5 | 5 | 5 | 6 | 6 |
| **3** | 6 | 5 | 5 | 6 | 4 | 6 |
| **4** | 6 | 6 | 6 | 6 | 6 | 6 |

**Table S5. Type Strain Genome Server (TYGS) analysis.** Genome-based taxonomic classification results.

| **Subject** | ***d_0_*** | **C.I. *d_0_*** | ***d_4_*** | **C.I. *d_4_*** | ***d_6_*** | **C.I. *d_6_*** | **Diff. G+C%** |
| --- | --- | --- | --- | --- | --- | --- | --- |
| *P. capeferrum* WCS358 | 91.9 | [88.9- 94.1] | 96.3 | [95.0- 97.3] | 94.8 | [92.8- 96.2] | 0.11 |
| *P. kermanshahensis* SWRI100 | 70.1 | 66.2- 73.8] | 41.6 | [39.1- 44.1] | 64.4 | 61.0- 67.6] | 0.34 |
| *A. frigidicola* CGMCC 1.9882 | 13.0 | [10.3- 16.3] | 36.1 | [33.7- 38.6] | 13.5 | [11.1- 16.2] | 4.95 |
| *P. asiatica* JCM 32716T | 52.1 | [48.6- 55.5] | 34.3 | [31.9- 36.8] | 47.4 | [44.4- 50.5] | 0.02 |
| *P. inefficax / P.* sp. JV551A3 | 53.1 | [49.6- 56.5] | 34.1 | [31.6- 36.6] | 48.1 | [45.1- 51.2] | 0.27 |
| *P. putida* NBRC 14164 | 58.9 | [55.3- 62.5] | 33.1 | [30.7- 35.6] | 52.1 | [49.0- 55.1] | 0.23 |
| *P. anuradhapurensis* RD8MR3 | 52.6 | [49.1- 56.0] | 32.8 | [30.3- 35.3] | 47.2 | [44.2- 50.2] | 0.87 |
| *P. kurunegalensis* RW1P2 | 55.5 | [51.9- 59.0] | 32.5 | [30.1- 35.0] | 49.3 | [46.2- 52.3] | 0.46 |
| *P. oryzicola R*D9SR1 | 52.5 | [49.1- 56.0] | 32.3 | [29.9- 34.8] | 47.0 | [44.0- 50.1] | 0.36 |
| *P. monteilii* DSM 14164 | 52.4 | [48.9- 55.8] | 32.2 | [29.8- 34.8] | 46.9 | [43.9- 49.9] | 1.07 |
| *P. plecoglossicida* DSM 15088 | 50.8 | [47.3- 54.2] | 31.8 | [29.4- 34.3] | 45.5 | [42.5- 48.5] | 0.41 |
| *P. sichuanensis* WCHPs060039 | 43.9 | [40.5- 47.3] | 30.0 | [27.6- 32.5] | 39.7 | [36.7- 42.8] | 1.31 |
| *P. urmiensis* SWRI10 | 44.6 | [41.2- 48.0] | 28.6 | [26.2- 31.1] | 39.7 | [36.7- 42.7] | 0.75 |
| *P. farsensis* SWRI107 | 39.3 | [35.9- 42.7] | 28.4 | [26.0- 30.9] | 35.7 | [32.8- 38.8] | 0.01 |
| *P. promysalinigenes* RW10S1 | 43.6 | [40.3- 47.1] | 28.1 | [25.7- 30.6] | 38.8 | [35.8- 41.9] | 1.94 |
| *P. shirazensis* SWRI56 | 37.4 | [34.1- 40.9] | 26.4 | [24.0- 28.9] | 33.7 | [30.8- 36.8] | 0.7 |
| **Strain** | | | **Identification result** | | | | |
| Pseudomonas sp. IsoF (CP072013.1) | | | *Pseudomonas capeferrum* | | | | |
| *d_0_: length of all HSPs divided by total genome length*  *d_4_: sum of all identities found in HSPs divided by overall HSP length*  *d_6_: sum of all identities found in HSPs divided by total genome length*  *C.I.: confidence intervals* | | | | | | | |

**Table S6**. List of predicted GEIs in *Pseudomonas capeferrum* IsoF

| Name | Gene start | Gene end | Island start | Island end | Size (bp) | GC%* | Type of island** | Program | Relevant features | Mobilization elements / insertion sites |
| --- | --- | --- | --- | --- | --- | --- | --- | --- | --- | --- |
| GEI 1 | *PisoF_00022* | *PisoF_00035* | 18595 | 32240 | 13613 | 57.1 | n.d. | 1 | Pyocin activator protein PrtN | Site-specific recombinase XerD |
| GEI 2 | *PisoF_00238* | *PisoF_00246* | 251855 | 272802 | 20950 | 59.5 | PAI | 1,2 | RHS repeat-associated core domain protein, type 1 fimbriae fimA | n.f. / tRNA |
| GEI 3 | *PisoF_00305* | *PisoF_00313* | 320588 | 330142 | 9555 | 63.5 | PAI | 1,2 | Multidrug resistance gene | IS3 family transposase |
| GEI 4 | *PisoF_00320* | *PisoF_00329* | 339970 | 354366 | 18340 | 55.2 | PAI | 1,2 | O-acetyltransferase OatA | n.f. |
| GEI 5 | *PisoF_00333* | *PisoF_00343* | 358931 | 372947 | 14000 | 60.2 | PAI-SI | 2 | Epimerase, glycosyl transferase, metallo-β-lactamase family protein | n.f. |
| GEI 6 | *PisoF_00790* | *PisoF_00793* | 850627 | 854251 | 3449 | 60.6 | PAI | 3 | **Acyl-homoserine-lactone synthase PpuI** | n.f. |
| GEI 7 | *PisoF_00931* | *PisoF_00945* | 1018861 | 1032560 | 13700 | 62.6 | n.d. | 1 | n.d. | IS3 family transposase |
| GEI 8 | *PisoF_00966* | *PisoF_00981* | 1056487 | 1078814 | 22329 | 53.8 | PAI-SI | 1,2 | Pyoverdine receptor, two subunits pilin, type 4 fimbrial assembly protein | Recombinase XerD, Prophage CP4-57 integrase / tRNA |
| GEI 9 | *PisoF_01084* | *PisoF_01097* | 1191647 | 1215802 | 27000 | 54.3 | PAI | 1,2 | n.d. | n.f. |
| GEI 10 | *PisoF_01248* | *PisoF_01270* | 1359836 | 1388824 | 28989 | 56.5 | PAI-SI | 1,2 | Restriction modification (RM) system | Integrase / tRNA |
| GEI 11 | *PisoF_01321* | *PisoF_01333* | 1456665 | 1469442 | 12777 | 60 | RI-SI | 2 | CreBC mediated β-lactam tolerance, putrecin importer, RHS repeat-associated core domain protein | n.f. |
| GEI 12 | *PisoF_01393* | *PisoF_01402* | 1530256 | 1542671 | 12413 | 51.4 | RI-SI | 1,2 | Pyoverdine receptor | Prophage CP4-57 integrase / tRNA |
| GEI 13 | *PisoF_01436* | *PisoF_01444* | 1580091 | 1592400 | 7083 | 62.9 | MI | 2 | Sulfonate uptake | n.f. |
| GEI 14 | *PisoF_01677* | *PisoF_01687* | 1864423 | 1874668 | 10176 | 61.3 | n.d. | 1,3 | Arsenic resistance proteins | n.f. |
| GEI 15 | *PisoF_02313* | *PisoF_02373* | 2569256 | 2636438 | 67179 | 58.8 | n.d. | 1,4 | **Type IVB secretion system Type I RM system** | Integrase / tmRNA |
| GEI 16 | *PisoF_02461* | *PisoF_02467* | 2737759 | 2744456 | 6698 | 66.7 | MI | 2 | n.d. | n.f. |
| GEI 17 | *PisoF_02751* | *PisoF_02758* | 3038696 | 3045696 | 7001 | 60.1 | RI-SI | 2 | Transcriptional Regulator CzcR | n.f. |
| GEI 18 | *PisoF_02942* | *PisoF_02956* | 3249656 | 3264121 | 14238 | 55.5 | PAI-RI-SI | 1,2 | Fimbrial units | n.f. / tRNA |
| GEI 19 | *PisoF_03023* | *PisoF_03032* | 3366463 | 3374467 | 8008 | 62.6 | PAI | 2 | YihY/virulence factor BrkB family protein | IS110 family transposase |
| GEI 20 | *PisoF_03413* | *PisoF_03416* | 3797876 | 3802950 | 4355 | 50.4 | n.d. | 1 | n.d. | Putative transposase OrfB / tRNA |
| Name | **Gene start** | **Gene end** | **Island start** | **Island end** | **Size (bp)** | **GC%*** | **Type of island**** | **Program** | **Relevant features** | **Mobilization elements / insertion sites** |
| GEI 21 | *PisoF_03555* | *PisoF_03561* | 3952460 | 3963228 | 10766 | 65.5 | PAI-RI-SI | 2 | Cobalt-zinc-cadmium resistance proteins | n.f. |
| GEI 22 | *PisoF_03601* | *PisoF_03609* | 4004244 | 4013806 | 9560 | 64.1 | MI | 2 | Sarcosine oxidase | n.f. |
| GEI 23 | *PisoF_03675* | *PisoF_03680* | 4084254 | 4089812 | 7370 | 62.2 | n.d. | 1,2 | n.d. | n.f. |
| GEI 24 | *PisoF_03696* | *PisoF_03719* | 4107953 | 4130440 | 22580 | 64.4 | n.d. | 1,2 | n.d. | n.f. |
| GEI 25 | *PisoF_03750* | *PisoF_03761* | 4171529 | 4184289 | 12761 | 61.2 | PAI-RI-SI | 1,2 | Amidase, Putrescine importer PuuP | n.f. |
| GEI 26 | *PisoF_03764* | *PisoF_03789* | 4187541 | 4219829 | 34402 | 57.9 | PAI-RI-SI | 1,2 | Aminoglycoside antibiotic resistance kinase | n.f. |
| GEI 27 | *PisoF_03825* | *PisoF_03830* | 4266556 | 4273812 | 8237 | 64.6 | SI-MI | 1,2 | n.d. | n.f. |
| GEI 28 | *PisoF_03852* | *PisoF_03859* | 4303868 | 4309697 | 7954 | 63.5 | PAI-RI-SI | 1,2 | n.d. | n.f. |
| GEI 29 | *PisoF_03917* | *PisoF_04079* | 4366596 | 4541954 | 175159 | 60.1 | PAI | 1,2,4 | Putative ICE with T4SS, Relaxase, T4CP | Transposase, putative prophage CPS-53 integrase / attL-R, tRNA |
| GEI 30 | *PisoF_04167* | *PisoF_04177* | 4630069 | 4638043 | 7973 | 57.8 | PAI-RI-SI | 2 | RTX-I toxin determinant B | n.f. |
| GEI 31 | *PisoF_04397* | *PisoF_04421* | 4861338 | 4889847 | 28507 | 59.4 | PAI-MI-RI-SI | 1,2 | n.d. | Transposase |
| GEI 32 | *PisoF_04476* | *PisoF_04481* | 4957315 | 5001450 | 44130 | 66.9 | n.d. | 1,3 | **Putisolvin cluster** | n.f. |
| GEI 33 | *PisoF_04608* | *PisoF_04618* | 5147801 | 5159219 | 11808 | 56.7 | n.d. | 1 | n.d. | n.f. |
| GEI 34 | *PisoF_04746* | *PisoF_04759* | 5305105 | 5318959 | 13855 | 58 | MI-RI | 1,2 | n.d. | Transposase |
| GEI 35 | *PisoF_04789* | *PisoF_04865* | 5351891 | 5441541 | 89022 | 53.7 | MI-RI-SI | 1,2 | Acetoin and 2,3-butanediol catabolism | IS66 family transposase ISPsy5 |
| GEI 36 | *PisoF_04919* | *PisoF_04925* | 5503915 | 5513210 | 10286 | 65.6 | n.d. | 1 | Virulence-associated OM protein Vir-90 | n.f. |
| GEI 37 | *PisoF_04957* | *PisoF_04976* | 5552508 | 5576256 | 36913 | 65.7 | PAI-MI | 1,2 | Colicin I receptor precursor | n.f. |
| GEI 38 | *PisoF_04998* | *PisoF_05036* | 5614495 | 5656341 | 41153 | 61.7 | RI | 1,2 | Colistin resistance protein EmrB | n.f. / tRNA |
| GEI 39 | *PisoF_05127* | *PisoF_05137* | 5737214 | 5744978 | 7761 | 55.2 | RI-SI | 1,2 | O-acetyltransferase OatA | n.f. / tRNA |
| GEI 40 | *PisoF_05297* | *PisoF_05307* | 5916243 | 5928891 | 12914 | 57.1 | n.d. | 1 | O-acetyltransferase OatA | Site-specific recombinase XerD |

* Coloured numbers indicate GC% difference of each GEI versus GC% of IsoF genome: > 3% in yellow, > 5% in orange, > 10% in dark red, < than 3% in black.

** Categorized by GIPSy. MI, Metabolic Island; PAI, Pathogenicity Island; RI, Resistance Island; SI, Symbiotic Island.

n.d.: not determined, n.f.: not found

1: IslandViewer4; 2: GIPSy; 3: Manual annotation; 4: ICEfinder.

**Table S7. Predicted phage regions in the genome of IsoF.**

| **Region** | **Size (bp)** | **Start** | **End** | **GC%** | **Completeness (score)** | **CDS*** | **Phage Hit CDS** | **Att**** | **Mobilization elements** |
| --- | --- | --- | --- | --- | --- | --- | --- | --- | --- |
| Reg. I | 19120 | 13214 | 32334 | 59.8 | Incomplete (40) | 12 | 8 | yes | Recombination endonuclease subunit D12 |
| Reg. II | 22061 | 2634970 | 2657031 | 62.1 | Incomplete (40) | 15 | 8 | yes | CP4-57 prophage; integrase |

*CDS: coding DNA sequence

**Att: site-specific attachment

**Table S8.** Genes predicted to code for IsoF adaptability and PGPR properties. Loci found within GEIs are indicated in red.

| **Gene** | **Locus** | **Product** | **GEI** |
| --- | --- | --- | --- |
| *czcA, czcB, czcC* | *PisoF_03557, PisoF_03556, PisoF_03555* | Cobalt/zinc/cadmium resistance proteins | 21 |
| *zntR, cadR* | *PisoF_01922, PisoF_01921* | Zinc transcriptional regulator, cadmium‑transporting ATPase | - |
| *arsC* | *PisoF_00471* | Arsenate reductase | - |
| *modA, modB, modC* | *PisoF_05117, PisoF_05118, PisoF_05119* | Molybdate transporters | - |
| *arsR, arsB, arsC* | *PisoF_01677, PisoF_01678, PisoF_01679* | Arsenic resistance proteins | 14 |
| *copZ, copA, copR* | *PisoF_01033, PisoF_01035, PisoF_01036* | Copper chaperone, copper-transporting ATPase, copper export regulator | - |
| *ttgC, ttgB, ttgA, ttgR* | *PisoF_02693 - PisoF_02006* | Toluene efflux pump, repressor | - |
| *mhpFE, mhpA, mhpD* | *PisoF_04003, PisoF_04004, PisoF_04421, PisoF_03927* | Partial cluster genes of 3-HTT degradation pathway | 29 31 |
| *sodB* | *PisoF_00718, PisoF_02648* | Fe superoxide dismutase | - |
| *sodC* | *PisoF_04647* | Cu/Zn superoxide dismutase | - |
| *katE, katG* | *PisoF_01586, PisoF_04880* | Catalase HPII | - |
| *katB, ydbD* | *PisoF_04011, PisoF_03952* | Catalase precursor, putative catalase | 29 |
| *ahpF, ahpC* | *05106, 05107* | Alkyl hydroperoxide reductase | - |
| *trpCDGE, trpIBA* | *PisoF_01220- PisoF_01224*  *PisoF_01622- PisoF_01625* | Tryptophan synthase related genes (Indole-3-acetic acid related genes) | - |
| *ddc, iaaM* | *PisoF_04983, PisoF_02081, PisoF_01075, PisoF_03886, PisoF_04218, PisoF_01000, PisoF_01271* | Tryptophan decarboxylase, tryptophan 2‑monooxygenase * | - |
| - | *PisoF_00738, PisoF_04212* | Auxin efflux carriers * | - |
| *acdS, acdA* | *PisoF_00157, PisoF_3667* | ACC deaminase | - |
| *acoABC, acoR* | *PisoF_04811, PisoF_04810, PisoF_04809, PisoF_04451* | Acetoin and 2,3-butanediol catabolism related genes, acetoin catabolism regulatory protein | 35 |

**Table S9. Predicted plastic degradation loci in IsoF.** All proteins were predicted to be secreted. Loci found within GEIs are indicated in red.

| **Gene** | **Locus** | **Product** | **Plastic type** | **E value** | **GEI** |
| --- | --- | --- | --- | --- | --- |
| *phaZ* | *PisoF_02059* | Polyhydroxyalkanoates depolymerase | Polyhydroxyalkanoates | 4.40 × 10⁻¹⁸⁷ | *-* |
| *estA* | *PisoF_02830* | Esterase | Polylactic acid | 4.10 × 10⁻¹³⁶ | *-* |
| *-* | *PisoF_00187* | Lipase | Polylactic Acid, Polyhydroxyalkanoate, Polyethersulfone, Polycaprolactone | 4.60 × 10⁻¹²⁸ | *-* |
| *-* | *PisoF_01648* | Polyethylene Glycol dehydrogenase | Polyethylene Glycol | 1.00 × 10⁻¹¹³ | *-* |
| *-* | *PisoF_02565* | Esterase | Polyethylene Terephthalate | 3.10 × 10⁻¹⁰² | *-* |
| *-* | *PisoF_03854* | Polyvinyl Alcohol dehydrogenase | Polyvinyl Alcohol | 1.40 × 10⁻⁹⁷ | 28 |
| *-* | *PisoF_03786* | Polyethylene Glycol aldehyde dehydrogenase | Polyethylene Glycol | 7.30 × 10⁻⁹¹ | 26 |
| *-* | *PisoF_03464* | Polyesterase | Polybutylene Adipate Terephthalate | 1.10 × 10⁻⁸⁸ | *-* |
| *hbdH* | *PisoF_04198* | Poly(3-hydroxyvalerate) dehydrogenase | Poly(3-hydroxyvalerate), Poly(3-hydroxybutyrate-co-3-hydroxyvalerate), Polyhydroxyalkanoate | 1.40 × 10⁻⁷² | *-* |
| *-* | *PisoF_03038* | Polylactic acid depolymerase | Polybutylene Succinate, Polybutylene Succinate Adipate, Polycaprolactone, Polyethersulfone, Polylactic Acid, Polyhydroxyalkanoate | 1.10 × 10⁻⁵⁶ | *-* |
| *-* | *PisoF_02776* | Hydrolase | Polylactic Acid, Poly(3-hydroxybutyrate-co-3-hydroxyvalerate), Polycaprolactone, Polybutylene Succinate Adipate, Polyethersulfone, Polyhydroxyalkanoate | 6.80 × 10⁻⁵² | *-* |
| *-* | PisoF_01068 | Esterase | Polylactic Acid, Polycaprolactone, Polybutylene Succinate Adipate | 2.20 × 10⁻¹⁹ | *-* |

**Table S10.** Prediction of virulence factors (VFs) in the *P. capeferrum* IsoF genome. VFs within GEIs are indicated in red.

| **VF class** | **IsoF prediction** | **Related genes** | **Virulence factors** | **GEIs** |
| --- | --- | --- | --- | --- |
| Adherence | **** | - | Flagella |  |
|  | *PisoF_00318; PisoF_00319; PisoF_00330; PisoF_00331; PisoF_00332; PisoF_00339; PisoF_00340* | - | LPS O-antigen  (*P. aeruginosa*) | 5 |
|  | **** | - | Type IV pili biosynthesis |  |
|  | **** | - | Type IV pili twitching motility related proteins |  |
| Antimicrobial activity | *PisoF_04438* | *phzH* | Phenazines biosynthesis |  |
| Antiphagocytosis | **** | - | Alginate biosynthesis and regulation |  |
| Iron uptake | *PisoF_03363* | *fptA* | Pyochelin receptor |  |
|  | *PisoF_00966; PisoF_01304; PisoF_01402; PisoF_01527; PisoF_01769; PisoF_02311; PisoF_02530; PisoF_03014; PisoF_03352; PisoF_03578; PisoF_03721; PisoF_04485; PisoF_04912; PisoF_04916* | *fpvA* | Pyoverdine receptors | 8 12 |
|  | **** | - | Pyoverdine |  |
| Quorum sensing | *PisoF_01646* | *hdtS* | Acylhomoserine lactone synthase |  |
|  | *PisoF_00790* | *ppuI* | N-(3-oxo-dodecanoyl)-L-homoserine lactone QS system | 6 |
|  | *PisoF_00792* | *ppuR* |  |  |
| Regulation | *PisoF_03409* | *gacA* | GacS/GacA two-component system |  |
|  | *PisoF_00466* | *gacS* |  |  |
|  | *PisoF_03121; PisoF_05121* | *csrA* | Carbon storage regulator A (*Legionella*) |  |
| Secretion systems | **** | - | T6SS |  |
|  | *PisoF_02264* | *hopAJ2* | *P. syringae* TTSS effectors |  |
|  | *PisoF_02504* | *hopJ1* |  |  |
| Toxin | *PisoF_00607* | *exlA* | Exolysin |  |
|  | *PisoF_00606* | *exlB* |  |  |
| Invasion | *PisoF_04898* | *ibeC* | Invasion of brain endothelial cells (*Escherichia*) |  |
| Lipid and fatty acid metabolism | *PisoF_03393* | *icl* | Isocitrate lyase (*Mycobacterium*) |  |
| Stress adaptation | *PisoF_04880* | *katG* | Catalase-peroxidase (*Mycobacterium*) |  |
|  | *PisoF_01152* | *katA* | Catalase (*Neisseria*) |  |
|  | *PisoF_04647* | *sodCI* | SodCI (*Salmonella*) |  |

** Genes or gene clusters required for these factors are found in IsoF

**Table S11.** Bacterial systems identified in IsoF by MacsyFinder. Loci found within GEIs are indicated in red.

| **System** | **CS (%)*** | **Homolog genes** | **Locus** | **GEIs** |
| --- | --- | --- | --- | --- |
| T1SS | 100 | *abc, mfp, omf* | *PisoF_00087, PisoF_00358, PisoF_00413, PisoF_00827, PisoF_00828, PisoF_00829, PisoF_00909, PisoF_01520, PisoF_01521, PisoF_02129, PisoF_02137, PisoF_02535, PisoF_02543, PisoF_02693, PisoF_03007, PisoF_03048, PisoF_03070, PisoF_03302, PisoF_03555, PisoF_03826, PisoF_04174, PisoF_04213, PisoF_04248, PisoF_04483, PisoF_04545, PisoF_04663, PisoF_04700, PisoF_04965, PisoF_04966, PisoF_04977, PisoF_04978, PisoF_04979, PisoF_05006, PisoF_05007* | 21  27  30  37  38 |
| T2SS | 69.2 | *gspD, gspE, gspF,  gspG, gspH, gspI,  gspK, gspK, gspO* | *PisoF_00611, PisoF_00612, PisoF_00613, PisoF_00614, PisoF_00615, PisoF_00616, PisoF_00617, PisoF_00621, PisoF_04252, PisoF_04253* | - |
| T4SS | 47.1 | *virb4, traI, traM,  traN, trap, traW, traY* | *PisoF_02314, PisoF_02315, PisoF_02317, PisoF_02322, PisoF_02326, PisoF_02337, PisoF_02353, PisoF_02354, PisoF_03979, PisoF_04027* | 15  29 |
| T5aSS | 100 | *PF03797* | *PisoF_01223, PisoF_04586, PisoF_05218* | - |
| T5bSS | 100 | Translocators | *PisoF_00606, PisoF_01048* | - |
| T6SS | 92.9 | *tssA, tssB, tssD, tssE,  tssF, tssG, tssH, tssI,  tssJ, tssK, tssL, tssM* | *PisoF_00988, PisoF_03592, PisoF_03601, PisoF_04294, PisoF_04452, PisoF_04938, PisoF_04939, PisoF_04941, PisoF_04943, PisoF_04944, PisoF_04945, PisoF_04947, PisoF_04948, PisoF_04949, PisoF_04950* | 22 |
| Type IVa pili | 88.2 | *pilB, pilC, pilT, pilQ,  pilA, pilD, pilE, pilV,  pilW, pilX, fimT, pilm,  pilN, pilO, pilP, pilZ* | *PisoF_00040, PisoF_00051, PisoF_00975, PisoF_00976, PisoF_00977, PisoF_01002, PisoF_01003, PisoF_01004, PisoF_01005, PisoF_01006, PisoF_01867, PisoF_01969, PisoF_01969, PisoF_01981, PisoF_01982, PisoF_01983, PisoF_01984, PisoF_02491, PisoF_02555, PisoF_02671, PisoF_03144, PisoF_03921, PisoF_04552, PisoF_04553, PisoF_04554, PisoF_04555, PisoF_04556, PisoF_04557, PisoF_04559* | 8  29 |
| Flagella | 100 | *sctJ, sctN, sctQ,  sctR, sctS, sctT,  sctU, sctV, flgB,  flgC, fliE* | *PisoF_01670, PisoF_01672, PisoF_03150, PisoF_03151, PisoF_03171, PisoF_03172, PisoF_03175, PisoF_03183, PisoF_03185, PisoF_03186, PisoF_03187, PisoF_03188, PisoF_03189* | - |

* CS: Predicted completeness Score (%)

| **Table S12.** T4SS effectors predicted using DeepSecE. | | | | | |
| --- | --- | --- | --- | --- | --- |
| **Protein** | **Uniprot code** | **Predicted activity** | **T4SS score** | **Similarity** | **GEI** |
| **PisoF_02350** | UPL06675.1 | Hypothetical protein | 0.9921 | YP_096517.1 | 15 |
| PisoF_00880 | UPL05243.1 | PA4642 family protein | 0.9843 | YP_153927.1 | - |
| PisoF_01503 | UPL05841.1 | Hypothetical protein DUF1841 | 0.9447 | YP_094294.1 | - |
| PisoF_05029 | UPL09321.1 | Hypothetical protein | 0.8729 |  | 38 |
| PisoF_05115 | UPL09404.1 | Hypothetical protein | 0.8659 | YP_094974.1 | - |
| PisoF_00971 | UPL05332.1 | AAA-like domain-containing protein | 0.8562 |  | 8 |
| PisoF_03573 | UPL07868.1 | OM barrel protein | 0.8491 | YP_094067.1 | 21 |
| PisoF_00667 | UPL05031.1 | DUF5064 family protein | 0.833 |  | - |
| **PisoF_02332** | UPL06657.1 | Proposed T4BSS immunity protein | 0.8263 |  | 15 |
| PisoF_00017 | UPL04403.1 | Putative formate dehydrogenase | 0.7737 | NP_882286.1 | - |
| PisoF_05290 | UPL09571.1 | DUF3106 hypothetical protein | 0.7366 | ACJ18454.1 | 40 |
| PisoF_01621 | UPL05948.1 | DUF883 family protein | 0.7337 | YP_096430.1 | - |
| PisoF_05156 | UPL09443.1 | Hypothetical protein | 0.7054 |  | 39 |
| **PisoF_02340** | UPL06665.1 | Tle1-like protein | 0.6885 | YP_094563.1 | 15 |
| PisoF_04182 | UPL08476.1 | Hypothetical protein | 0.6814 | WP_010947043.1 | - |
| PisoF_02056 | UPL06383.1 | Phasin family protein | 0.6814 |  | - |
| PisoF_03893 | UPL08188.1 | Hemerythrin, domain-containing protein | 0.6738 | ACJ19636.1 | - |
| PisoF_03445 | UPL07741.1 | DUF6388 family protein | 0.6713 | YP_094134.1 | - |
| PisoF_03776 | UPL08071.1 | DUF4435 domain-containing protein | 0.6495 |  | 26 |
| PisoF_00970 | UPL05331.1 | Hypothetical protein | 0.6239 | YP_001972768.1 | 8 |
| PisoF_03942 | UPL08236.1 | Hypothetical protein | 0.6191 |  | 29 |

n.d.: not determined

**3. Supplementary References**

1.	Chen W, Kuo TT. A simple and rapid method for the preparation of Gram-negative bacterial genomic DNA. *Nucleic Acids Res* 1993;**21**:2260. https://doi.org/10.1093/nar/21.9.2260

2.	Walker BJ et al. Pilon: An integrated tool for comprehensive microbial variant detection and genome assembly improvement. *PLoS One* 2014;**9**:e112963. https://doi.org/10.1371/journal.pone.0112963

3.	Seemann T. Prokka: Rapid prokaryotic genome annotation. *Bioinformatics* 2014;**30**:2068–2069. https://doi.org/10.1093/bioinformatics/btu153

4.	Carver TJ et al. ACT: the Artemis comparison tool. *Bioinformatics* 2005;**21**:3422–3423. https://doi.org/10.1093/BIOINFORMATICS/BTI553

5.	Krumsiek J, Arnold R, Rattei T. Gepard: a rapid and sensitive tool for creating dotplots on genome scale. *Bioinformatics* 2007;**23**:1026–1028. https://doi.org/10.1093/BIOINFORMATICS/BTM039

6.	Cumsille A et al. GenoVi, an open-source automated circular genome visualizer for bacteria and archaea. *PLoS Comput Biol* 2023;**19**. https://doi.org/10.1371/JOURNAL.PCBI.1010998

7.	Purtschert-Montenegro G et al. *Pseudomonas putida* mediates bacterial killing, biofilm invasion and biocontrol with a type IVB secretion system. *Nat Microbiol* 2022;**7**:1547–1557. https://doi.org/10.1038/s41564-022-01209-6

8.	Butaite E et al. Siderophore cheating and cheating resistance shape competition for iron in soil and freshwater *Pseudomonas* communities. *Nat Commun* 2017;**8**. https://doi.org/10.1038/s41467-017-00509-4

9.	Loftie-Eaton W et al. Draft genome sequence of *Pseudomonas* sp. nov. H2. *Genome Announc* 2016;**3**. https://doi.org/10.1128/genomeA.00241-15

10.	Geels FP, Schippers B. Reduction of yield depressions in high frequency potato cropping soil after seed tuber treatments with antagonistic fluorescent *Pseudomonas* spp. *J Phytopathol* 1983;**108**:207–214. https://doi.org/10.1111/j.1439-0434.1983.tb00580.x

11.	Devescovi G et al. A siderophore peptide synthetase gene from plant-growth-promoting *Pseudomonas putida* WCS358. *Syst Appl Microbiol* 2001;**24**:321–330. https://doi.org/10.1078/0723-2020-00063

12.	Wu X et al. Comparative genomics and functional analysis of niche-specific adaptation in *Pseudomonas putida*. *FEMS Microbiol Rev* 2011;**35**:299–323. https://doi.org/10.1111/j.1574-6976.2010.00249.x

13.	Matilla MA et al. Complete genome of the plant growth-promoting rhizobacterium *Pseudomonas putida* BIRD-1. *J Bacteriol* 2011;**193**:1290. https://doi.org/10.1128/JB.01281-10

14.	Nelson KE et al. Complete genome sequence and comparative analysis of the metabolically versatile *Pseudomonas putida* KT2440. *Environ Microbiol* 2002;**4**:799–808. https://doi.org/10.1046/j.1462-2920.2002.00366.x

15.	Wang W et al. Genetic mapping of highly versatile and solvent-tolerant *Pseudomonas putida* B6-2 (ATCC BAA-2545) as a ‘superstar’ for mineralization of PAHs and dioxin-like compounds. *Environ Microbiol* 2021;**23**:4309–4325. https://doi.org/10.1111/1462-2920.15613

16.	Hanahan D. Studies on transformation of *Escherichia coli* with plasmids. *J Mol Biol* 1983;**166**:557–580. https://doi.org/10.1016/S0022-2836(83)80284-8

17.	Simon R, Priefer U, Pühler A. A broad host range mobilization system for in vivo genetic engineering: Transposon mutagenesis in gram negative bacteria. *Bio/Technology* 1983;**1**:784–791. https://doi.org/10.1038/nbt1183-784

18.	Steidle A et al. Visualization of N-acylhomoserine lactone-mediated cell-cell communication between bacteria colonizing the tomato rhizosphere. *Appl Environ Microbiol* 2001;**67**:5761–5770. https://doi.org/10.1128/AEM.67.12.5761-5770.2001

19.	Cárcamo-Oyarce G et al. Quorum sensing triggers the stochastic escape of individual cells from *Pseudomonas putida* biofilms. *Nat Commun* 2015;**6**. https://doi.org/10.1038/ncomms6945

20.	Conway HF et al. *Pseudomonas aureofaciens* Kluyver and phenazine alpha-carboxylic acid, its characteristic pigment. *J Bacteriol* 1956;**72**:412–417. https://doi.org/10.1128/jb.72.3.412-417.1956

21.	Bernal P et al. The *Pseudomonas putida* T6SS is a plant warden against phytopathogens. *ISME J* 2017;**11**:972–987. https://doi.org/10.1038/ismej.2016.169

22.	Choi KH, Schweizer HP. mini-Tn7 insertion in bacteria with single attTn7 sites: Example *Pseudomonas aeruginosa*. *Nat Protoc* 2006;**1**:153–161. https://doi.org/10.1038/nprot.2006.24

23.	Koch B, Jensen LE, Nybroe O. A panel of Tn7-based vectors for insertion of the *gfp* marker gene or for delivery of cloned DNA into Gram-negative bacteria at a neutral chromosomal site. *J Microbiol Methods* 2001;**45**:187–195. https://doi.org/10.1016/S0167-7012(01)00246-9

24.	Flannagan RS, Linn T, Valvano MA. A system for the construction of targeted unmarked gene deletions in the genus *Burkholderia*. *Environ Microbiol* 2008;**10**:1652–1660. https://doi.org/10.1111/j.1462-2920.2008.01576.x

25.	Figurski DH, Helinski DR. Replication of an origin-containing derivative of plasmid RK2 dependent on a plasmid function provided in trans. *Proc Natl Acad Sci U S A* 1979;**76**:1648–1652. https://doi.org/10.1073/pnas.76.4.1648
